# Gut Microbiota of Obese Children Influences Inflammatory Mucosal Immune Pathways in the Respiratory Tract to Influenza Virus Infection: Optimization of an Ideal Duration of Microbial Colonization in a Gnotobiotic Pig Model

**DOI:** 10.1128/spectrum.02674-21

**Published:** 2022-05-17

**Authors:** Sankar Renu, Loic Deblais, Veerupaxagouda Patil, Jennifer Schrock, Dipak Kathayat, Vishal Srivastava, Ninoshkaly Feliciano-Ruiz, Yi Han, Anikethana Ramesh, Yashavanth S. Lakshmanappa, Shristi Ghimire, Santosh Dhakal, Gireesh Rajashekara, Gourapura J. Renukaradhya

**Affiliations:** a Center for Food Animal Health, Department of Animal Sciences, Ohio Agricultural Research and Development Center, Wooster, Ohio, USA; Oklahoma State University, College of Veterinary Medicine

**Keywords:** human fecal microbiota, obese, healthy, influenza virus, gnotobiotic pigs, immune maturation, inflammation

## Abstract

The impact of obesity on the human microbiota, immune maturation, and influenza virus infection has not been yet established in natural host animal models of influenza. In this study, gnotobiotic (Gn) pigs were colonized with human fecal microbiota (HFM) of obese (oHFM) or healthy lean (hHFM) children and infected at different periods (2-, 3-, and 5-weeks post-transplantation) using a zoonotic influenza virus strain. The infected oHFM pigs were characterized by lower levels of Firmicutes (*Lactococcus, Lactobacillus, Turicibacter*, and Streptococcus) and Actinobacteria (*Bifidobacterium*), which was associated with higher levels of Proteobacteria (Klebsiella), Bacteroidetes, and Verrucomicrobia (*Akkermansia*) compared with the infected hHFM group (*P* < 0.01). Furthermore, these genera significantly correlated with the expression of immune effectors, immune regulators, and inflammatory mediators, and displayed opposite trends between oHFM and hHFM groups (*P* < 0.01). The lymphoid and myeloid immune cell frequencies were differently modulated by the oHFM and hHFM colonization, especially apparent in the 5-weeks HFM colonized piglets. In addition, oHFM group had higher pro-inflammatory cytokines (IL-6, IL-12, TNF-α, and IFNγ) gene expression in the respiratory tract compared with the hHFM colonized pigs was detected. In conclusion, pigs colonized for longer duration, established oHFM increased the immune maturation favoring the activation of inflammatory mediators, however, the influenza virus load remained comparable with the hHFM group. Further, a longer duration of microbial colonization (5 weeks) may be required to reveal the impact of microbiome on the host immune maturation and susceptibility to influenza virus infection in the humanized Gn pig model.

**IMPORTANCE** The diversity of gut microbiome of obese people differs markedly from that of lean healthy individuals which, in turn, influences the severity of inflammatory diseases because of differential maturation of immune system. The mouse model provides crucial insights into the mechanism(s) regulating the immune systems mediated by the gut microbiota but its applicability to humans is questionable because immune cells in mice are poorly activated in microbiota humanized mice. Several important strains of *Bifidobacterium*, *Lactobacillus*, and *Clostridium* fails to colonize the murine gut. Thus, understanding the role of certain important commensal gut bacterial species influences upon health and disease, a suitable large animal model like pig that supports the growth and colonization of most of the important human gut bacteria and possess comparable immunology and physiology to humans is beneficial to improve health.

## INTRODUCTION

Trillions of indigenous microorganisms present in the host are responsible for maintaining good health as well as precipitating diseases if they are dysregulated (1). Microorganisms colonized during the first year of life are the foremost deciding factor for rest of the life in human health (2). The indigenous microbiota influences the normal development and function of the mucosal immune system as well as inhibiting the colonization of non-native pathogenic microorganisms (3). Influenza is the most prevalent seasonal viral infection in humans and animals (4). Influenza virus infection causes acute respiratory inflammation in humans and pigs, responsible for high fever, body aches, and fatigue (5, 6). To mitigate the severity of influenza, research has been emerging in recent years on gut microbiota transplantation therapy due to low efficacy of traditional vaccines in evading severity of disease and virus transmission (7). Studies have revealed that a healthy gut microbiome activates the inflammasome via toll-like receptors (8), and the microbial metabolites triggers type-I interferon signaling (9) thus, protecting the host against influenza virus infection (10).

Obesity-induced inflammatory mediators have an impact on human health, as well as is a common risk factor for many chronic human illnesses (cardiovascular diseases, metabolic syndromes, and cancer) that result in high mortality (11, 12). Obesity can exacerbate influenza virus infection (i.e., deep lung viral replication, progression of pneumonia, and continued higher viral shedding) (13, 14). Obesity has been recognized as a risk factor for increased disease severity and mortality in infected individuals with the pandemic 2009 H1N1 influenza A virus (13). Studies demonstrated that an imbalanced gastrointestinal microbiome plays a key role in gut permeability and development of obesity (15–18), via activation of the lipopolysaccharide-mediated inflammatory innate immune responses (19). Characteristic changes in the gut microbiome composition have been reported, such as an increase in potential butyrate producing Firmicutes to the detriment of other short chain fatty acid (SCFA) producing Bacteroidetes. Thereby, the Firmicutes:Bacteroidetes (F:B) ratio could be considered as the hallmark for the development of obesity; and great discrepancies are reported about this topic (20–23). Several studies demonstrated that obese individuals harbored greater gut microbiome diversity and higher F:B ratio compared with healthy lean individuals (24). Specific Firmicutes bacteria (i.e., *Blautia hydrogenotorophica*, Coprococcus catus, Eubacterium ventriosum, *Lactobacilus leuteri*, Ruminococcus bromii, Ruminococcus obeum, and Staphylococcus) were closely associated with obesity, while lean individuals were characterized by higher abundance in Bacteroides faecichinchillae and *thetaiotaomicron*, Blautia wexlerae, *Bulleidia*, Clostridium bolteae, Flavonifractor plautii, Methanobrevibacter smithii, *Oribacterium*, and *Veillonella* (24–27) However, these trends were only observed in the lower intestinal tract and were influenced by the gender and biomass index of the participant (26, 28, 29). In addition, the gut microbiota influenced inflammatory factors by modulating the secretion of inflammatory cytokines, and IL-6 and TNF-α are the biomarkers associated with inflammation and obesity (30).

**FIG 1:**
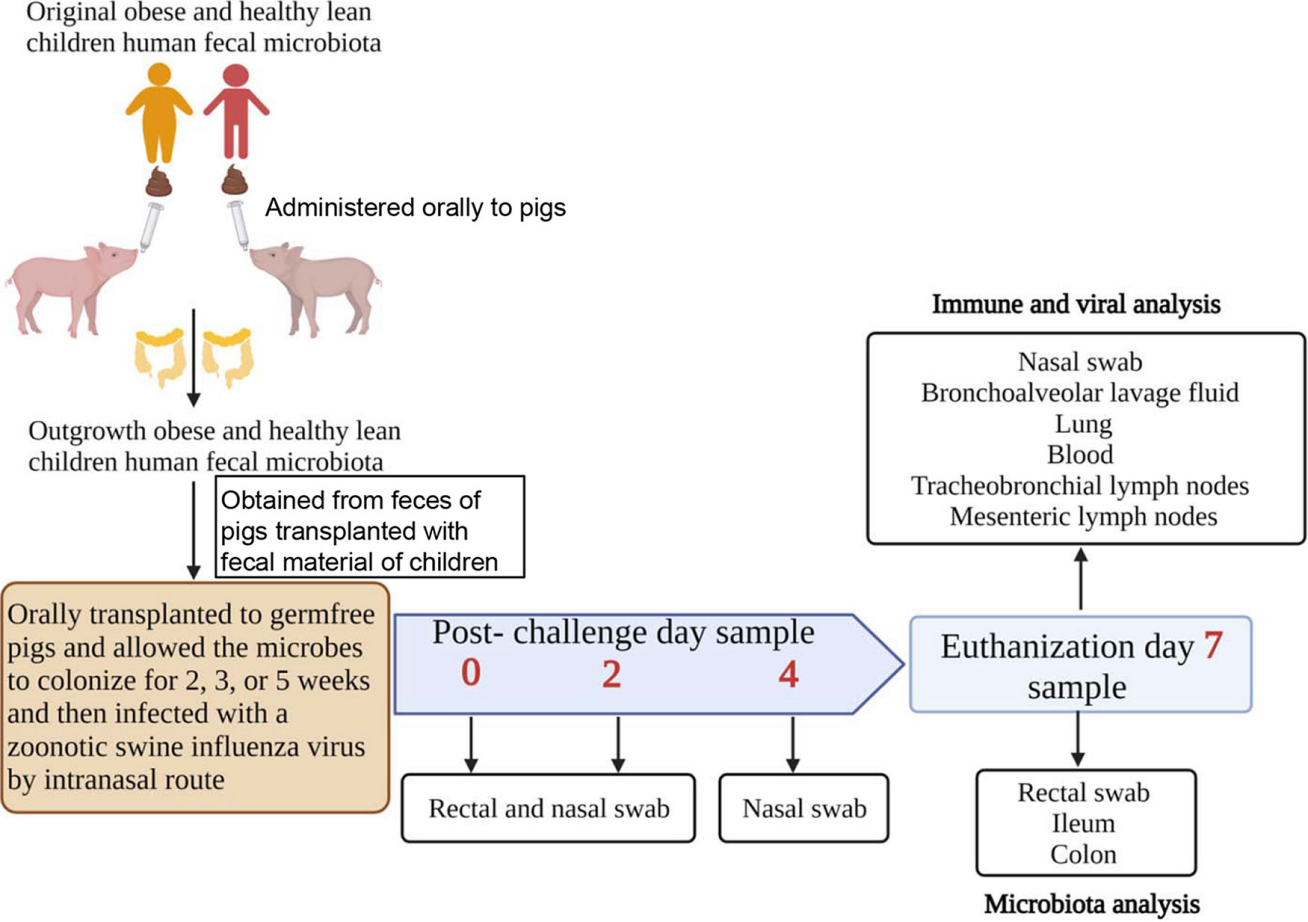
Schematic illustration of the experimental overview, longitudinal sampling time points, and details of the analysis. Initially, Gn pigs were transplanted with fecal material of obese and healthy lean children and colonized human microbiota in pig intestines (outgrowth) was collected and transplanted to all the experimental Gn pigs.

Selection of a suitable animal model is very important to understand the impact of the microbiome on host immune development and susceptibility to diseases (31). Swine are an appropriate animal model to understand the influenza virus infection over rodents, as pig is the natural host for influenza, and its anatomical, immunological, physiological, and genetic compositions are much similar to humans (32–34). In our earlier study, we successfully used germ-free gnotobiotic (Gn) pigs to transplant rural (Amish) and urban (non-Amish) infants’ fecal microbiota. The transplanted pigs harbored comparable microbial diversity and composition compared with the original infant fecal inoculum (31). Furthermore, the transplanted microbiome differentially modulated the development of mucosal immune system in the gut and the maturation of immune cells in the mucosa associated lymphoid tissue in humanized pigs (31). In this study, we transplanted obese HFM (oHFM) and healthy lean children HFM (hHFM) into Gn pigs to understand how the host microbiome composition modulates the host immune system maturation, especially in the respiratory tract. Further, humanized pigs were infected with a virulent swine influenza virus at different times (2, 3, and 5 weeks after HFM transplantation) (i) to assess the importance of the microbiome diversity and abundance to viral infection and (ii) correlate maturation of the microbiome and immune system in the host. This approach facilitated the identification of optimum duration required post-transplantation for the microbiome/immune maturation in obese humanized pigs that impacted the susceptibility to influenza virus infection. In Fig. 1, we have shown a schematic illustration of the experiment overview, longitudinal sampling and details of the analysis.

## RESULTS

### The HFM transplant did not substantially influence the influenza virus load in humanized pigs.

**FIG 2:**
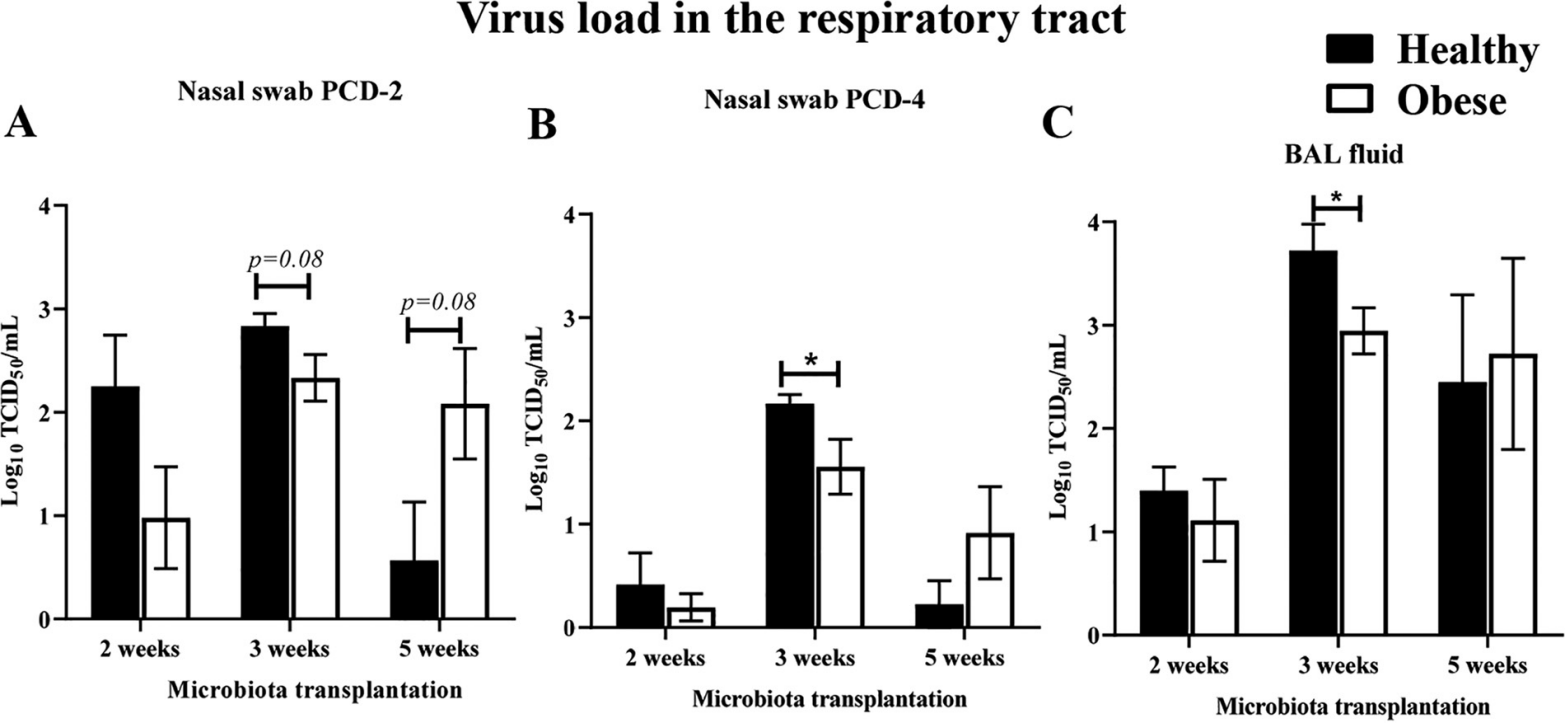
Influence of diverse gut microbiota on influenza virus infection. Gnotobiotic piglets were inoculated with healthy lean and obese outgrowth HFM and at 2-, 3-, and 5-weeks post transplantation challenged with SwIAV H1N1-OH7. Samples collected at different postchallenge day (PCD) were analyzed for the challenge virus load in (A) nasal swab at PCD 2; (B) nasal swab at PCD 4; and (C) BAL fluid at PCD 7. Data are presented as the mean of 4 to 6 pigs ± SEM analyzed using *unpaired t test*. Asterisks denote significant difference (*, *P* < 0.05).

The obese and healthy outgrowth HFM transplanted pigs were allowed to colonize with microbes for 2, 3, or 5 weeks and then infected with influenza virus. Pigs had a mild fever (up to 102°F) only on the first 2 days postinfluenza infection irrespective of type of microbiota transplantation and the duration of microbial colonization. We did not observe any flu-associated visible clinical signs other than the fever. The virus titers were checked in the nasal swab at postchallenge day (PCD) 2 and 4, and in bronchoalveolar lavage fluid (BAL) fluid at PCD 7 (Fig. 2A to C). In the nasal swab samples, the virus titers were comparable between the groups of pigs at PCD-2 irrespective of different lengths (2, 3, or 5 weeks) of colonization of oHFM and hHFM microbes (Fig. 2A), and were significantly (*P* < 0.05) reduced at PCD-4 in oHFM compared with hHFM colonized pigs infected only after 3 weeks of transplantation (Fig. 2B). Similarly, in BAL fluid of oHFM transplanted piglets infected with the virus after 3 weeks of microbial colonization (but not at two other time points) observed significantly reduced virus load (*P* < 0.05) compared with hHFM cohort group (Fig. 2C).

### Obese HFM transplantation increased the pro-inflammatory cytokine gene expression in the draining lymph nodes.

**FIG 3:**
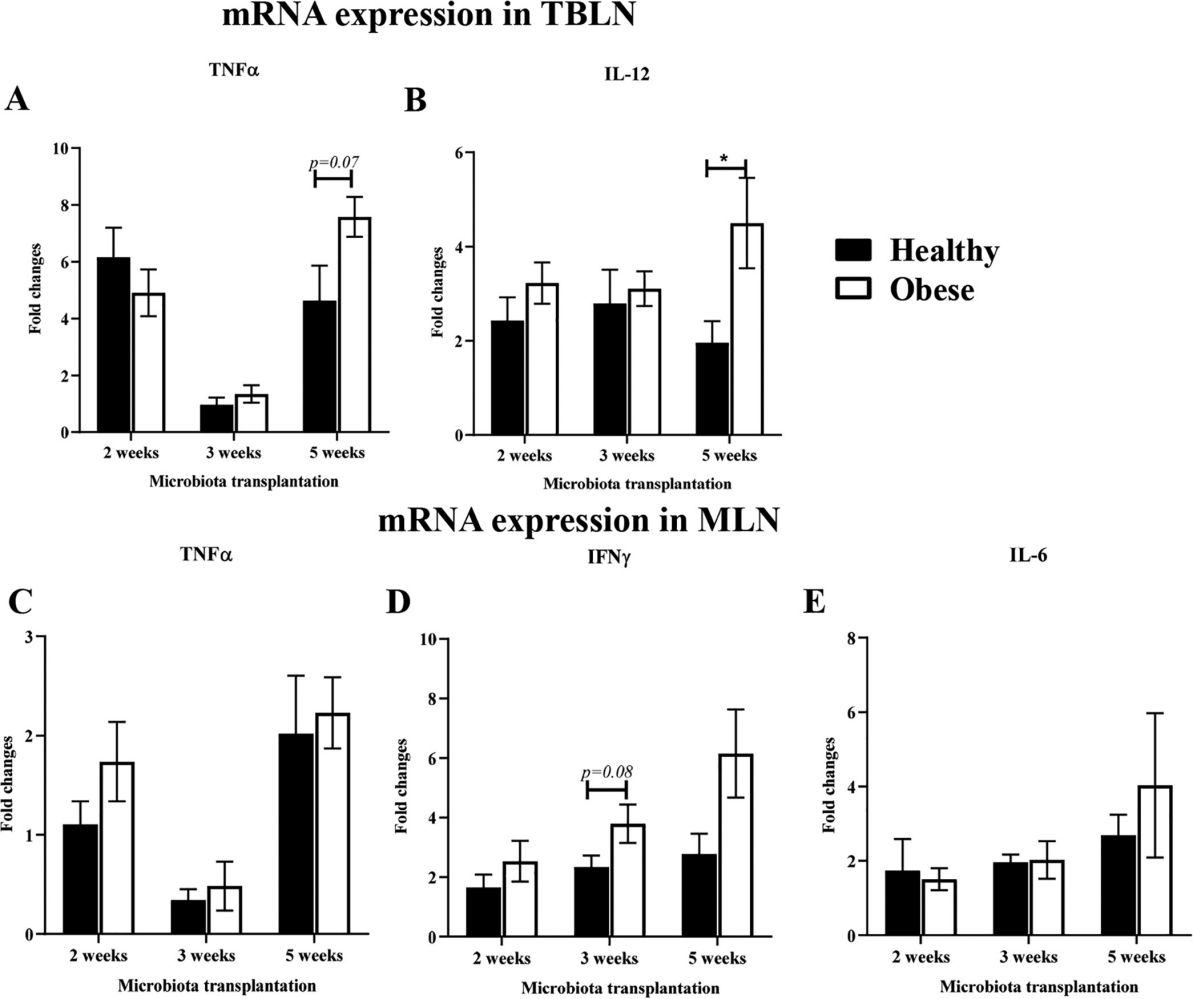
Activation of pro-inflammatory cytokines expression in the mucosal lymph nodes of pigs mediated by gut microbiota and influenza virus infection. Gnotobiotic piglets were inoculated with healthy lean and obese outgrowth HFM and at 2-, 3-, and 5-weeks post transplantation challenged with SwIAV H1N1-OH7. At PCD 7 animals were euthanized and RNA extracted from tissues were analyzed for mRNA expression by qRT-PCR in TBLN (A) TNF-α and (B) IL-12; and in MLN (C) TNF-α; (D) IFNγ; and (E) IL-6. Data are presented as the mean of 4 to 6 pigs ± SEM analyzed using *unpaired t test*. Asterisks denote significant difference (*, *P* < 0.05).

In the oHFM transplanted pigs at early time points (2 and 3 weeks) postcolonization did not induce detectible changes in the expression of most of the analyzed pro-inflammatory cytokines’ mRNA in tracheobronchial lymph nodes (TBLN) and mesenteric lymph nodes (MLN) (Fig. 3). However, prolonged colonization of obese HFM for up to 5 weeks modulated the immune maturation especially at the distant respiratory tract with significant upregulation in the expression of IL-12 mRNA in TBLN of pigs following the influenza virus infection (Fig. 3B). Further, though statistically not significant, increased trends in the expression of other inflammatory cytokines such as TNF-α in TBLN in pigs infected after 5 weeks of oHFM transplantation, and IFNγ (but not TNF-α and IL-6) in MLN of animals infected after 3 weeks of oHFM transplantation compared with hHFM colonized animals (Fig. 3A to D and E).

### Lymphoid and myeloid immune cells population are differentially activated by the influence of diverse gut microbiota of children in colonized pigs.

**FIG 4:**
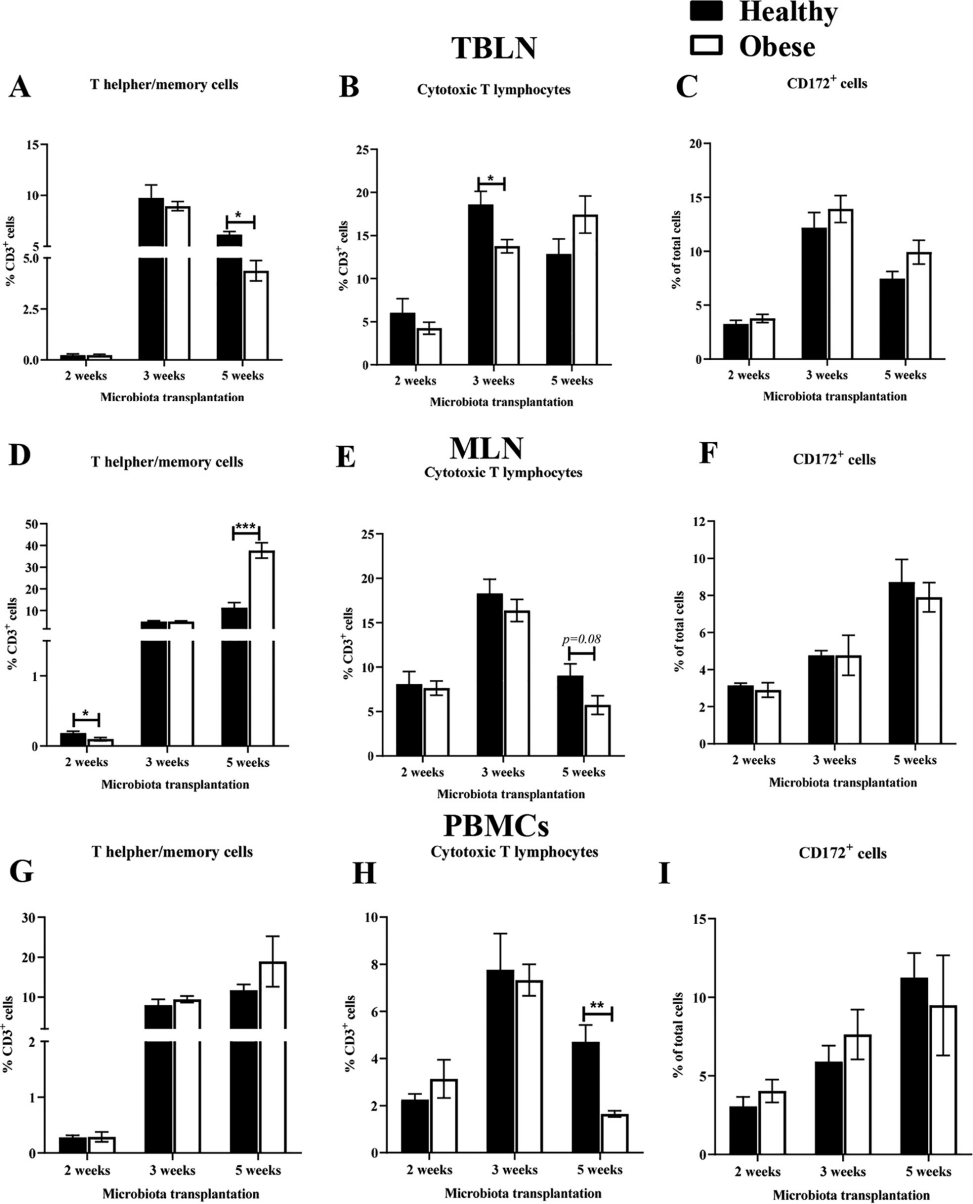
Modulation in the population of lymphoid and myeloid immune cells in the mucosal and systemic sites of pigs influenced by colonized HFM and influenza virus. Gnotobiotic piglets were inoculated with healthy lean and obese outgrowth HFM and at 2-, 3-, and 5-weeks post transplantation challenged with SwIAV H1N1-OH7. At PCD 7 animals were euthanized and MNCs isolated from TBLN, MLN, and blood (PBMCs) were immunostained and analyzed for the frequency of different lymphoid and myeloid cells by flow cytometry. (A, D, G) T helper/memory cells; (B, E, H) Cytotoxic T lymphocytes; and (C, F, I) CD172^+^ myeloid cells. Data are presented as the mean of 4 to 6 pigs ± SEM analyzed using *unpaired t test*. Asterisks denote significant difference (*, *P* < 0.05, **, *P* < 0.01, and ***, *P* < 0.001).

The healthy and obese HFM colonization in Gn piglets induced differential activation of immune cells in terms of relative abundance of important T cell subsets population in both systemic (blood) and mucosal (respiratory and intestinal) lymphoid tissues (Fig. 4). In particular, T helper/memory cells in TBLN of hHFM colonized pigs had significantly (*P* < 0.05) higher frequency compared with their oHFM cohorts, and this was observed only in extended duration (5 weeks) of HFM colonized pigs following influenza virus infection (Fig. 4A). While in hHFM colonized pigs at 3 weeks post-transplantation significantly (*P* < 0.05) increased frequency of cytotoxic T cells compared with their oHFM cohorts was detected (Fig. 4B). In the MLN of oHFM colonized pigs only at 5-weeks post-transplantation observed a significantly (*P* < 0.05) increased T helper/memory cells frequency and decreased trend in the population of cytotoxic T cells (Fig. 4D and E). Further representing the immune cells modulation at systemic site, peripheral blood mononuclear cells (PBMCs) of oHFM 5-weeks colonized animals had a significantly (*P* < 0.05) decreased frequency of cytotoxic T cells compared with their hHFM cohorts with no significant alteration in T helper/memory cells frequency (Fig. 4G). Meanwhile, the myeloid immune cells frequency was comparable between hHFM and oHFM transplanted animals at all three evaluated post-transplantation weeks in both systemic as well as mucosal lymphoid tissues sites (Fig. 4C and F and I).

### Microbiome composition of the original HFM and outgrowth HFM used for the pig’s transplantations.

The original HFM collected from healthy lean children (original hHFM) was composed of five major phyla such as Proteobacteria (49.6%), Firmicutes (26.4%), Bacteroidetes (15.7%), Actinobacteria (5.5%), and Euryarchaeota (1.3%). Same phyla were detected in the original HFM collected from obese children (original oHFM), but in different proportions: Bacteroidetes (54.7%), Firmicutes (38.4%), Actinobacteria (5.7%), Proteobacteria (0.8%), and Euryarchaeota (0.01%). Only Verrucomicrobia was detected in both the groups with a relative abundance below 1% (0.3% in original hHFM and 0.01% in original oHFM groups).

The efficiency of HFM transplantation and the similarity of the microbial compositions of the original human and Gn pig fecal outgrowth samples was confirmed by 16S metagenomic (V4 to V5 region sequencing) analysis based on open OTU. Similar microbiota composition (>99%) was observed when the original HFM (hHFM and oHFM) was compared with its corresponding pig outgrowth HFM, at the genus level (Fig. S1). *Bacteroides*, Klebsiella, *Enterococcus*, *Ruminococcus*, *Anoxybacillus*, *Prevotella*, and *Corynebacterium* were the major genus detected in both original HFM (relative abundance > 1%). Similarly, >95% microbiome composition was observed when the pig outgrowth HFM was compared with the original HFM (Fig. S1). The original HFM samples had *Prevotella* (10.2%), *Faecalibacterium* (1.4%), *Phascolarctobacterium* (0.4%), *Megamonas* (0.2%), *Dialister* (0.1%), and these OTUs were not detected in the allied outgrowth samples (Fig. S1). Likewise, Klebsiella (1.6%), *Lactococcus* (0.4%), and *Lactobacillus* (0.4%) were detected in the outgrowth oHFM samples but not in the associated original oHFM samples (Fig. S1).

### Impact of HFM transplant and infection time upon microbiome composition of humanized pigs before and after influenza challenge.

Gn pigs were transplanted with hHFM or oHFM and challenged with influenza at 2, 3, or 5 weeks post-transplantation. Fecal and nasal swabs were collected at PCD 0, 2, and 7; and respiratory (BAL fluid and lung), intestinal (ileum, and colon), and MLN tissue samples were collected on PCD 7. A total of 369 samples were processed for 16S rRNA V4-V5 variable region sequencing. After processing and taxonomic assignment with the latest SILVA v138.1 reference database; 14,969,576 sequences were obtained (average of 39,085 ± 34,771 sequences per samples).

**FIG 5:**
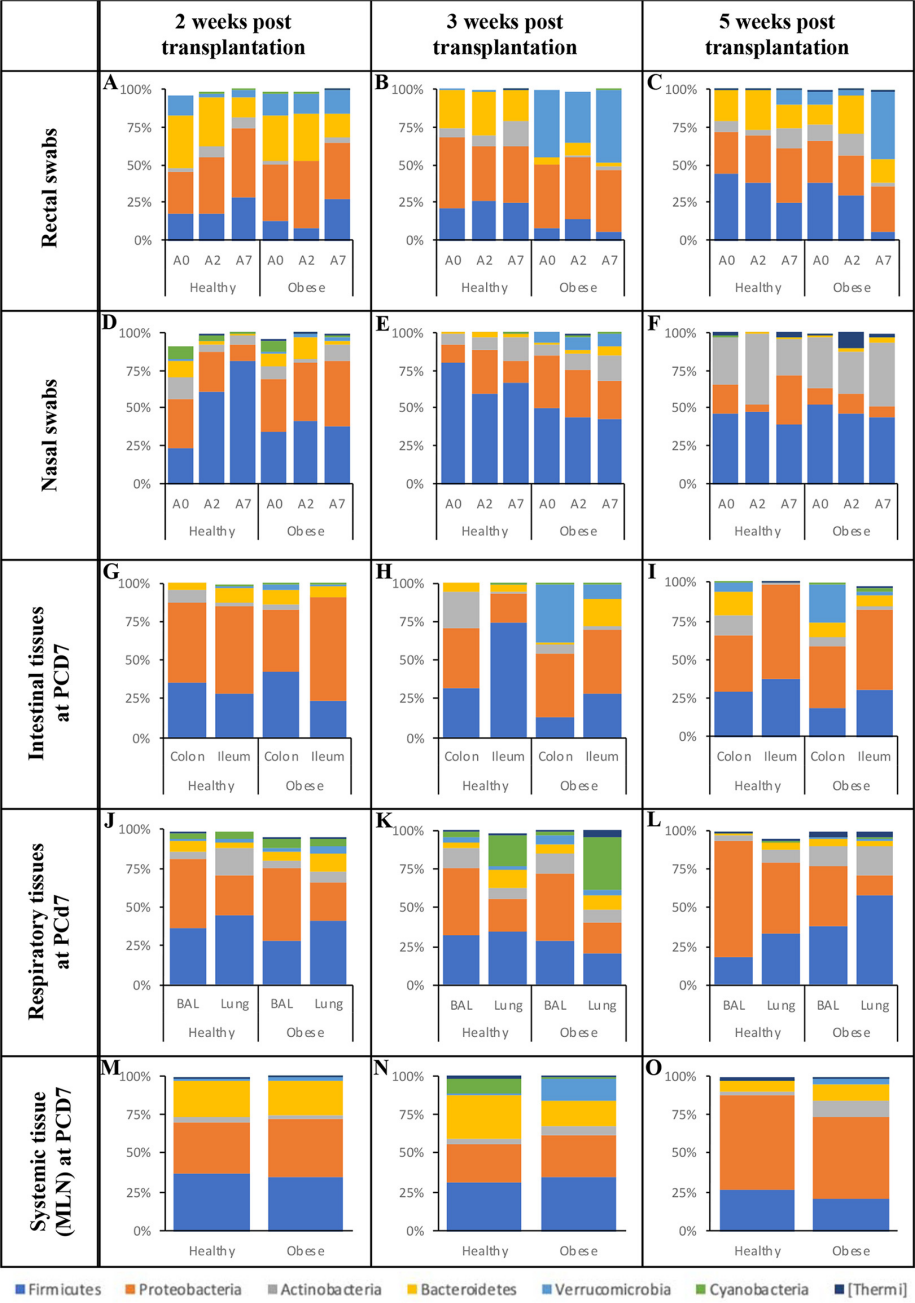
Microbiota composition at the phylum level in fecal, intestinal, and systemic tissues across the three challenging experiments. Relative abundance in rectal (A to C) and nasal (D to F) swabs at postchallenging day 0 (A0), 2 (A2), and 7 (A7). Relative abundance in intestinal (ileum and colon; G to I), respiratory (lung and BAL; J to L) and systemic tissues (MLN; M to O) at postchallenging day 7 (PCD 7). Obese and healthy: pigs transplanted with an obese or healthy human fecal microbiota (HFM), respectively. 2-, 3-, and 5-weeks post-transplantation: maturation time between HFM transplantation and influenza challenge. NS, nasal swab; RS, rectal swab; BAL, bronchoalveolar lavage fluid; MLN, mesenteric lymph node. Details about the significant differences in relative abundance (from phylum to species levels) detected between hHFM and oHFM for a designated incubation time post-transplantation, time point post influenza challenge and sample type are displayed in Table S3.

**FIG 6:**
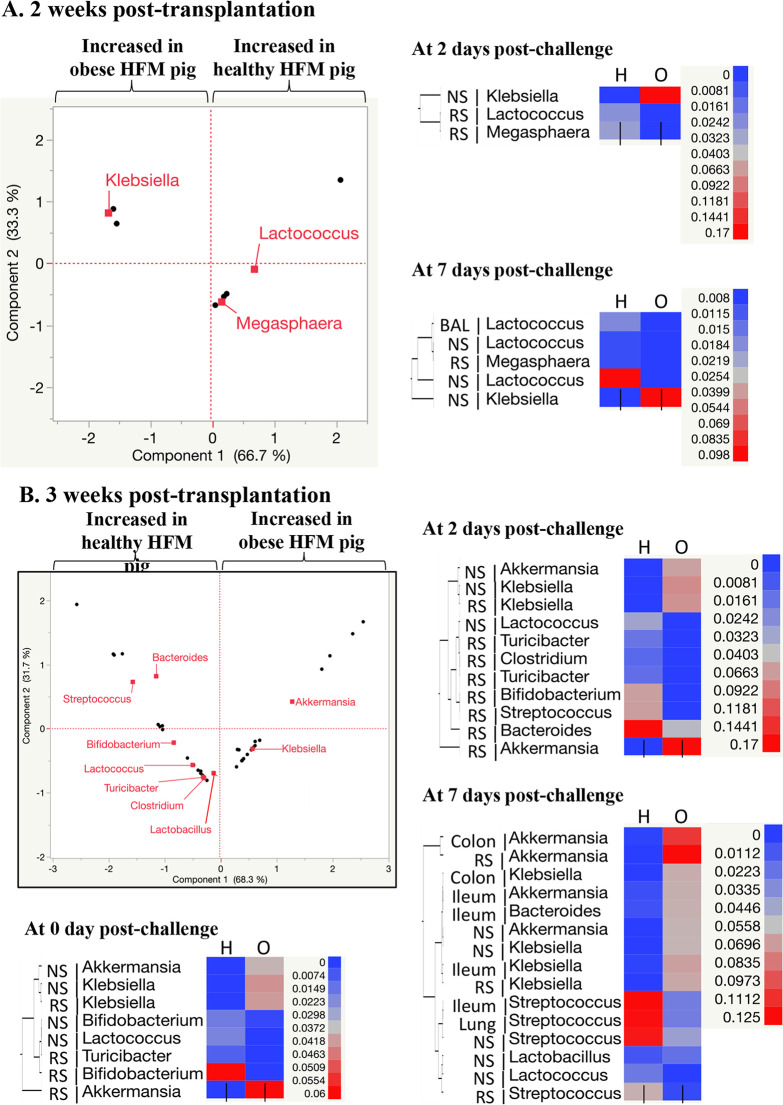
Differences in microbiota composition at the genus level between healthy and obese HFM pig samples. Microbiome differences observed in the 2-weeks (A) and 3-weeks (B) post-transplantation experiments. Data presented in the principal-component analysis plot were generated based on the associated heatmap data presented for each time point (0, 2, and 7 days PCD). The PCA displays the significant differences detected between oHFM and hHFM, across all time points and sample types. Only predominant OTUs (relative abundance > 1%) showing significant differences (*P* < 0.01) between the healthy and obese groups are displayed in these figures. O and H: pigs transplanted with an obese or healthy human fecal microbiota (HFM), respectively. The color of the cell is associated with the relative abundance of the OTU in the designated tissue and time point. NS, nasal swab; RS, rectal swab; BAL, bronchoalveolar lavage fluid; MLN, mesenteric lymph node. Details about the significant differences in relative abundance (from phylum to species levels) detected between hHFM and oHFM for a designated incubation time post transplantation, time point post influenza challenge and sample type are displayed in Table S3.

Overall, the majority of the samples displayed significant lower alpha diversity (Shannon index) in hHFM pig samples (*n* = 15/33) compared with oHFM pig samples (*n* = 8/33) across the three post transplantation periods (Table S2; *P* < 0.01). This trend was more pronounced in the respiratory tissues (80%; nasal swab, BAL fluid and lung) compared with the intestinal tissues (60%; rectal swab, ileum, and colon). Differences between hHFM and oHFM samples were also observed with the beta diversity (Table S2). The microbiota (weighted uniFrac) of the oHFM group clustered away from the hHFM group for the designated sample types, time points, and experiments (*P* < 0.01; entropy r2 > 0.75; Table S2). Overall, based on the relative abundance data, the microbiota composition at 3 weeks post-transplantation displayed the most differences between the hHFM and oHFM groups in the different sample types and time points compared with the 2- and 5-weeks post-transplantation (Fig. 5 and 6; Table S3), while the microbiota composition at 5-weeks post-transplantation showed the least differences between the hHFM and oHFM groups.

### Impact of HFM transplant and influenza infection on rectal swab microbiome over time.

The rectal swabs of oHFM and hHFM pigs were collected at PCD 0, 2, and 7 from pigs colonized for different lengths of time (2-, 3-, and 5-weeks post-transplantation; Fig. 5A to C). We observed Firmicutes (5% to 44%) and Proteobacteria (26% to 46%) constituted most of the microbiota, followed by Bacteroidetes (3% to 35%), Actinobacteria (0% to 16%), and Verrucomicrobia (0% to 48%).

At 2-weeks post-transplantation, *Lactococcus* was significantly higher in the hHFM group in the rectal swab (5,287.3-fold) at PCD 2 compared with the oHFM group (*P* < 0.01; Fig. 6A and Table S3). *Megasphaera* was only detected in the hHFM group in the rectal swab (1.8%) at PCD 2, and significantly higher in the rectal swab of the hHFM (3,508.2-fold) at PCD 7 compared with the oHFM group (*P* < 0.01; Fig. 6A and Table S3). Other low abundance OTUs (< 1%) such as *Eggerthella* (6.2-fold) was significantly higher in the hHFM group at PCD 7 compared with the oHFM group (*P* < 0.01; Table S3).

At 3-weeks post-transplantation, most of the differences at the phylum level between the hHFM and oHFM groups were observed with the Verrucomicrobia, which was associated with significantly lower levels of *Akkermansia* in the hHFM group (104.6- and 224.5-fold between PCD 0 to PCD 7), compared with the oHFM group (*P* < 0.01; Fig. 5, 6B; Table S3). In addition, the hHFM group harbored significantly higher abundance in Actinobacteria at PCD 0 (74.7-fold) and PCD 2 (42.5-fold) compared with the oHFM group (Fig. 5B; Table S3), which was associated with the higher abundance of *Bifidobacterium* (139.4- and 57.1-fold at PCD 0 and PCD 2, respectively), *Collinsella* (17.5-fold at PCD 2; *P* < 0.01; Fig. 6B; Table S3). *Eggerthella* was only detected in the hHFM group at PCD 0 and PCD 2 (relative abundance between 0.01 and 0.02%; *P* < 0.01; Table S3). Further, hHFM group harbored significantly higher abundance in Bacteroidetes at PCD 2 (3.5-fold) compared with the oHFM group (Fig. 5B; Table S3), which was associated with an increased abundance of *Bacteroides* (4.4-fold) and *Odoribacter* (35.5-fold; *P* < 0.01; Fig. 6B; Table S3). The hHFM group were also characterized by significantly higher abundance of Firmicutes at PCD 7 (4.5-fold) compared with the oHFM group (Fig. 5B; Table S3), which was associated with the increased abundance of *Coprococcus* (8.0-fold), *Clostridium* (12.2-fold), *Dorea* (64-fold), *Lactococcus* (147.3-fold), Streptococcus (12.3-fold), and *Turicibacter* (64-fold; *P* < 0.01; Fig. 6B and Table S3). Despite the lack of significant differences in Proteobacteria between the hHFM and oHFM group in the rectal swabs (Fig. 5B), Klebsiella (1,948.1-fold at PCD 0, 2,096.6-fold at PCD 2, and 660.8-fold at PCD 7), *Citrobacter* (62-fold at PCD 0, 59.7-fold at PCD 2, and 22.3-fold at PCD 7), *Erwinia* (35.8-fold at PCD 0 and 35.2-fold at PCD 2) were significantly lower in the hHFM group compared with the oHFM group (*P* < 0.01; Fig. 6B; Table S3). *Sutterella* was only detected in the hHFM group at PCD 7 (relative abundance of 0.4%; *P* < 0.01; Table S3). *Trabulsiella* was only detected in the hHFM group between PCD 0 and PCD 7 (relative abundance between 0.001% and 0.003%; Table S3).

At 5-weeks post-transplantation, no difference was detected at the phylum level between the hHFM and oHFM groups (Fig. 5C). However, the abundance of Blautia (23.7-fold) and Holdemania (27.1-fold) were higher in the hHFM group at PCD 0 compared with the oHFM group (*P* < 0.01; Table S3).

### Impact of HFM transplant and influenza on nasal microbiome.

Overall, Firmicutes (23% to 81%) and Proteobacteria (6% to 44%) constituted most of the microbiota, followed by Bacteroidetes (1% to 14%), Actinobacteria (3% to 47%), Verrucomicrobia (0% to 9%), Cyanobacteria (0% to 9%) and Thermi (0% to 11%) in the nasal swabs of oHFM and hHFM at PCD 0, 2, and 7 from pigs colonized for different lengths of time (2-, 3-, and 5-weeks post-transplantation; Fig. 5D to F).

At 2-weeks post-transplantation, the Firmicutes population significantly increased in the hHFM group at PCD 2 (61%) and PCD 7 (81%) compared with PCD 0 (23%; *P* < 0.01), while its abundance was similar in the oHFM group between PCD 0 and PCD 7 (18% to 29%; *P* > 0.05). The abundance of Firmicutes was significantly higher (81%) and the abundance of Proteobacteria was significantly lower (12%) at PCD 7 in the hHFM group compared oHFM group (29% and 44%, respectively; *P* < 0.01; Fig. 5D and Table S3). The increase of Firmicutes in the nasal swabs of the hHFM group between PCD 0 and PCD 7 was associated with the abundance of Staphylococcus over time, which became significantly higher at PCD 7 (58 ± 12.8%) in the hHFM group compared with the oHFM group (18.5 ± 6.5%; *P* < 0.01; Table S3). Further, the reduction in Proteobacteria in the hHFM group was associated with significantly lower levels in Klebsiella at PCD 2 (2,500-fold) and at PCD 7 (1,375-fold) compared with the oHFM group (*P* < 0.01; Fig. 6A and Table S3). Other low abundance OTUs (< 1%) such as *Odoribacter* and *Oscillospira* (85.4- and 37.2-fold, respectively) at PCD 2, and *Bacillus* (6.2-fold) at PCD 7 were significantly higher in the hHFM group compared with the oHFM group (*P* < 0.01; Table S3).

At 3-weeks post-transplantation, the rectal swab samples, oHFM group had significantly lower Verrucomicrobia associated with lower *Akkermansia* (*P* < 0.01; Fig. 5E, 6B; Table S3). The hHFM group was characterized by significantly higher abundance of *Bifidobacterium* (4.6-fold at PCD 0), *Dorea* (16.2-fold at PCD 2), Escherichia (4.7-fold at PCD 2), Streptococcus (3.0-fold at PCD 7), and *Lactococcus* (7.4-fold at PCD 0, 71.3-fold at PCD 2, and 113.6-fold at PCD 7) compared with the oHFM group (*P* < 0.01; Fig. 6B; Table S3). Similar trends were observed with other low abundant OTUs (< 1%) such as *Megasphaera* (3.6-fold at PCD 0), *Turicibacter* (10.2-fold at PCD 2) and *Eggerthella* (5.3-fold at PCD 2 and 5.7-fold at PCD 7; *P* < 0.01; Table S3). On the other hand, the hHFM group was characterized by significantly lower abundance of Klebsiella (629-fold at PCD 0, 143.5-fold at PCD 2, and 41-fold at PCD 7) compared with the oHFM group (*P* < 0.01; Fig. 6B; Table S3). Similar trends were observed with other low abundant OTUs (< 1%) such as *Erwinia* (20.7-fold at PCD 0), *Veilonella* (262.1-fold at PCD 2), *Blautia* (6.6-fold at PCD 7), and *Lactobacillus* (2.2-fold at PCD 7; *P* < 0.01; Table S3).

At 5 weeks post-transplantation, no differences were detected at the phylum level between the hHFM and oHFM groups (Fig. 5F). However, the abundance of *Ruminococcus* (4.6-fold) was lower in the hHFM group at PCD 0 compared with the oHFM group (*P* < 0.01; Table S3).

### Impact of HFM transplant and influenza on intestinal microbiome.

Overall, Firmicutes (13% to 74%) and Proteobacteria (19% to 67%) constituted most of the microbiota, followed by Bacteroidetes (0% to 17%), Actinobacteria (1% to 24%), Verrucomicrobia (0% to 37%), and Cyanobacteria (0% to 2%) in the intestinal tissues (ileum and colon) of oHFM and hHFM at PCD 7 from pigs colonized for different lengths of time (2-, 3-, and 5-weeks post-transplantation; Fig. 5G to I).

At 2-weeks post-transplantation, no difference was observed at the phylum level (Fig. 5G). However, *Lactococcus* was significantly higher in the hHFM group in the ileum (440.7-fold) compared with the oHFM group (*P* < 0.01; Table S3).

At 3-weeks post-transplantation, the trends observed in the rectal swabs (Fig. 5B) between PCD 0 and PCD 7 were also detected in the intestinal tissues (ileum and colon) at PCD 7 (Fig. 5H; *P* < 0.01). More precisely, the hHFM group was characterized by significantly lower abundance of Verrucomicrobia (i.e., *Akkermansia*; 13.9-fold and 304.2-fold in the ileum and colon, respectively) at PCD 7 compared with oHFM group (*P* < 0.01; Fig. 5H and 6B; Table S3). Further, the hHFM group harbored significantly higher abundance of Firmicutes (i.e., Streptococcus [4.8-fold]) in the ileum at PCD 7 compared with oHFM group (*P* < 0.01; Fig. 6B; Table S3). On the other hand, the hHFM group had significantly lower abundance of Klebsiella in both ileum (65.4-fold) and colon (221.1-fold) at PCD 7 compared with oHFM group (*P* < 0.01; Fig. 6B; Table S3). Similar trends were observed with other low abundant OTUs (< 1%) such as *Citrobacter* (57.6-fold and 16.5-fold, in ileum and colon, respectively) and *Erwinia* (34.9-fold and 14.2, in ileum and colon, respectively; *P* < 0.01; Table S3). At 5-weeks post-transplantation, no difference between the hHFM and the oHFM groups was detected in the intestinal tissues at PCD 7 (Fig. 5I).

### Impact of HFM transplant and influenza on respiratory microbiome.

Overall, Firmicutes (18% to 58%) and Proteobacteria (14% to 76%) constituted most of the microbiota, followed by Bacteroidetes (1% to 12%), Actinobacteria (3% to 19%), Verrucomicrobia (0% to 5%), Cyanobacteria (0% to 33%) and Thermi (0% to 5%) in respiratory tissues of oHFM and hHFM at PCD 7 from pigs colonized for different lengths of time (2-, 3-, and 5-weeks post-transplantation) (Fig. 5J to L). Likewise in the intestines, in 2-weeks post-transplanted hHFM group BAL fluid sample significantly higher *Lactococcus* was detected (*P* < 0.01; Fig. 6A; Table S3).

At 3-weeks post-transplantation (Fig. 5K), the trends observed in the nasal swabs (Fig. 5E) between PCD 0 and PCD 7 were also detected in the respiratory tissues (BAL fluid and lung) at PCD 7 (*P* < 0.01; Fig. 5K). The hHFM group was characterized by significantly higher levels of Streptococcus (4.8-fold in the lung) at PCD 7 compared with the oHFM groups (*P* < 0.01; Fig. 6B; Table S3). Opposite trend was observed with other low abundance OTUs (< 1%) such as *Veillonella* which was significantly lower (21.3-fold) in the BAL fluid of hHFM at PCD 7 compared with the oHFM groups (*P* < 0.01; Table S3).

At 5-weeks post-transplantation, no difference between the hHFM and the oHFM groups was detected in the respiratory tissues at PCD 7 (Fig. 5L).

### Impact of HFM transplant and influenza on systemic microbiome.

Overall, Firmicutes (21% to 38%) and Proteobacteria (25% to 61%) constituted most of the microbiota, followed by Bacteroidetes (6% to 28%), Actinobacteria (2% to 11%), Verrucomicrobia (0% to 14%), Cyanobacteria (0% to 9%), and Thermi (0% to 2%) in MLN of oHFM and hHFM at PCD 7 from pigs colonized for different length of time (2-, 3-, and 5-weeks post-transplantation; Fig. 5M to O).

At 2-weeks post-transplantation, no difference between the hHFM and the oHFM groups was detected in the MLN at PCD 7 at the phylum (Fig. 5M); However, *Lactococcus* was only detected in the hHFM group in the MLN (0.6%) at PCD 7 (*P* < 0.01; Fig. 6A; Table S3). At 3-weeks post-transplantation (Fig. 5N), no difference between the hHFM and the oHFM groups was detected in the MLN at PCD 7 at the phylum or genus/species level. At 5-weeks post-transplantation, no difference between the hHFM and the oHFM groups was detected in the MLN at PCD 7 at the phylum (Fig. 5O); However, low abundance OTUs (< 1%) such as *Bilophila* was significantly lower (25.5-fold) in the MLN of the hHFM group at PCD 7 compared with the oHFM group (*P* < 0.01; Table S3).

### Correlations of microbiome composition between sample types for a designated HFM type and length of colonization.

A multivariate analysis was used to determine if the gut microbiome composition impacts the microbiome in other tissues (systemic and respiratory tissues). Overall, higher microbiome composition similarity profile (weighted uniFrac) was observed at PCD 7 between the intestinal (colon, ileum, and rectal swab) and systemic tissues (MLN) compared with the respiratory tissues (BAL, lung and nasal swab; *P* < 0.01; Fig. S2A). These data suggest that the gut microbiome likely has more impact the systemic tissues’ microbiome composition than the respiratory’ microbiome in this model. In addition, it was observed that the abundance of specific OTUs correlated between tissue types (total of 61 OTUs; *n* = 27 to 31 OTUs per each colonization experiment); however, these correlations were highly affected by the colonization length (2-, 3-, and 5-weeks post transplantation; Fig. S2B) and the type of HFM transplant (hHFM versus oHFM; Fig. S2C; *P* < 0.01). The relative abundance of most OTUs (*n* = 51/61) positively correlated between tissue types. Most of the positively and negatively correlated OTUs belonging to Proteobacteria (45%), Firmicutes (28%), and Actinobacteria (13%). These data suggest that both microbiome composition and colonization duration may modulate the translocation, persistence, and abundance of specific bacteria between tissues. Details about the OTUs (at the genus level) showing correlation between tissue types are described in Fig. S2C.

### Correlations between the immune parameters and microbiota of gut and respiratory tissues.

**FIG 7:**
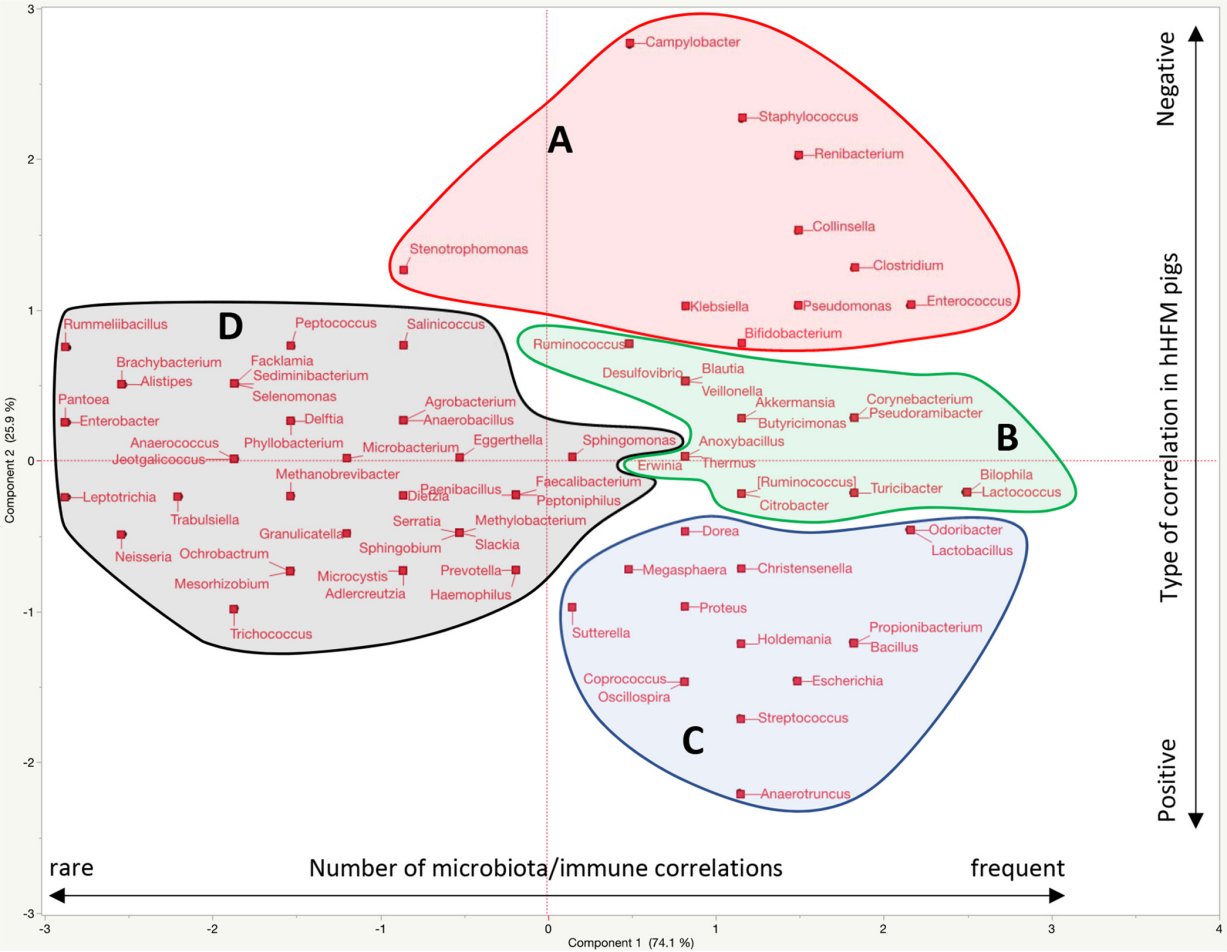
Distribution of the microbiota collected from intestinal (rectal swab, colon, and ileum) and respiratory (nasal swab, lung, and BAL fluid) tissues of the healthy human fecal microbiota (hHFM) pig group at 7 days postchallenge (PCD 7), across the three experiments (2-, 3-, and 5-weeks post-transplantation) based on its correlations between the immune responses (immune regulators, immune effectors, and inflammatory responses). Clusters A (*n* = 10) and C (*n* = 15) indicates OTUs frequently negatively or positively correlated with the immune parameters, respectively. Cluster B (*n* = 16) indicates the OTUs frequently negatively and positively correlated with the immune parameters. Clusters D (*n* = 40) indicates the OTUs not frequently correlated with the immune parameters. Inflammatory responses (TNF-α, IL-6, and IFNγ), immune effectors (CD3+CD4+CD8α + β- and CD3+CD4-CD8α + β+T cells), and immune regulators (IL-12 and CD3-CD172^+^).

The microbiota of the intestinal (rectal swab, ileum, colon, and MLN) and respiratory (nasal swab, lung, and BAL fluid) tissues collected at PCD 7 were correlated with the immune parameters (immune effectors, immune regulators, and inflammatory responses). A global representation of the correlations between the microbiota composition and immune parameters in the intestinal and respiratory tissues of hHFM group across the three experiments is presented in Fig. 7. A total of 18 microbiota/immune combinations were tested across the three experiments (infection at 2-, 3-, and 5-weeks post-transplantation). Overall, 59.4% of the OTUs detected at the genus level (82/138) were significantly correlated (r^2^ > 0.5 or r^2^ < −0.5; *P* < 0.01) with at least one of the immune parameters and displayed opposite trends between the hHFM and oHFM groups. An equivalent correlation was observed with the immune parameters between the intestinal and respiratory tissues across the three experiments (Fig. 7). However, 5-weeks post-transplantation displayed highest number of correlations between the microbiota and the immune parameters (approximately 1.4-fold) and the highest mean r^2^ (absolute mean r^2^ = 0.59) compared with 2- and 3-weeks post-transplantation (absolute mean r^2^ = 0.54 and 0.55, respectively). The opposite trends were observed in the oHFM group. The microbiota was separated into four major clusters (A to D) based on the number and the type of correlations with immune parameters. Cluster A was composed of 10 OTUs (e.g., Campylobacter, Staphylococcus, and *Renibacterium*) frequently positively correlated with the immune parameters in the hHFM group. Cluster C was composed of 15 OTUs (e.g., *Anaerotruncus*, Streptococcus, *Oscillospira*, *Coprococcus*, and Escherichia) frequently negatively correlated with the immune parameters in the hHFM group. Cluster B was composed of 16 OTUs (e.g., *Bilophila*, *Lactococcus*, *Turicibacter*, *Pseudoramibacter*, *Corynebacterium*, *Citrobacter*, *Ruminococcus*, *Akkermansia*, and Butyricimonas) frequently positively and negatively correlated with the immune parameters in the hHFM group. Cluster D (*n* = 40) was composed of OTUs rarely positively and negatively correlated with the immune parameters in the hHFM group.

**FIG 8:**
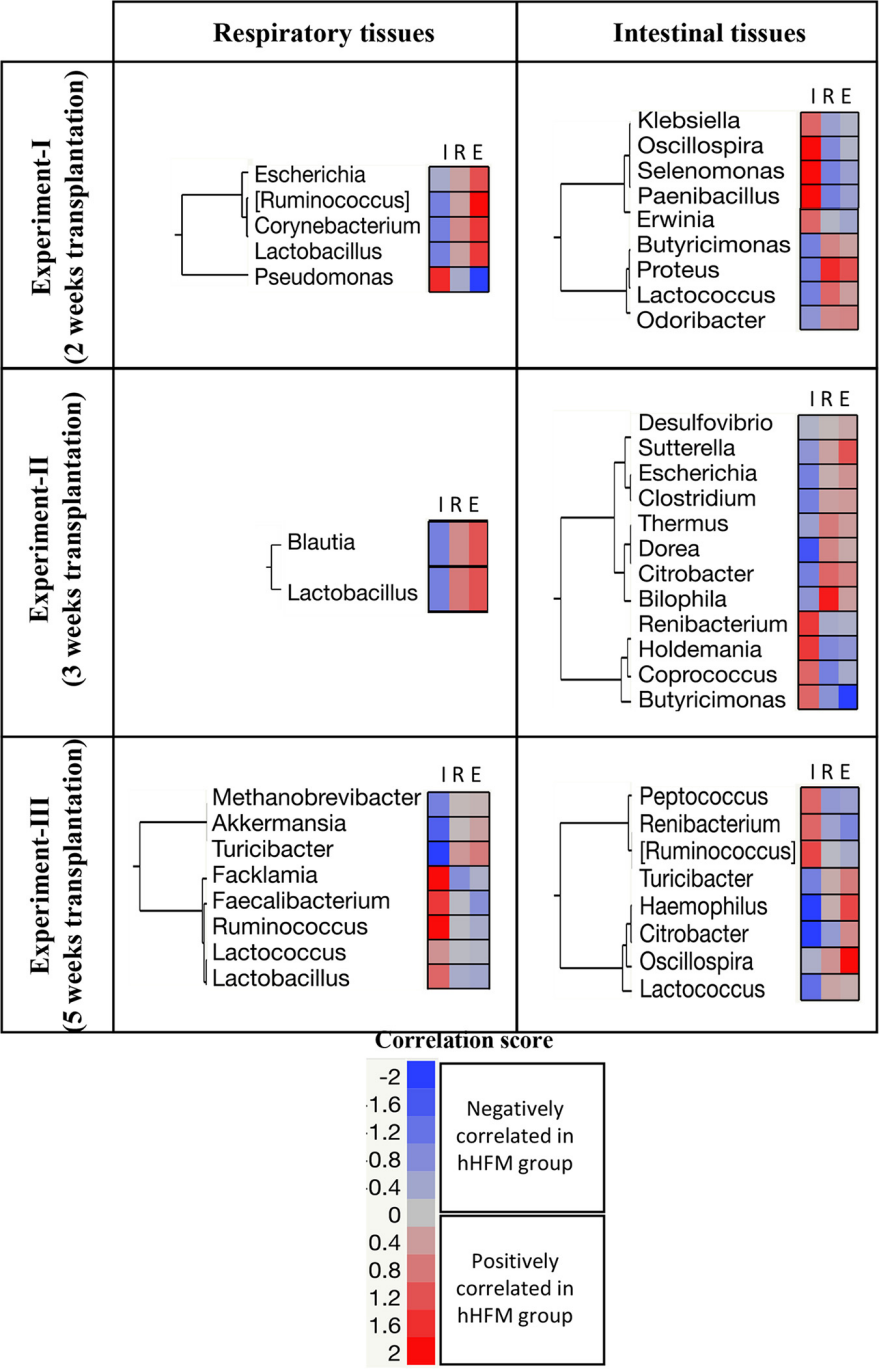
Microbiome-immune response correlations in intestinal and systemic tissues across the three experiments. The two-way clustering plots were generated using only significant correlations (*P* > 0.01 and r^2^ lower than −0.25 or higher than 0.25) at PCD 7. The color of the cells is associated with the type of correlations between the designated OTU and immune parameter. Blue and red cells: negative or positive correlation, respectively, between the designated OTU and immune parameter in the healthy HFM group. The opposite trend was observed in the obese HFM group. Obese and healthy group: pigs transplanted with an obese or healthy human fecal microbiota (HFM), respectively. I, inflammatory responses (TNF-α, IL-6, and IFNγ); E, immune effectors (CD3+CD4+CD8α + β- and CD3+CD4-CD8α + β+T cells); and R, immune regulators (IL-12 and CD3-CD172^+^).

For this study, we focused on OTUs in the hHFM group that were either negatively or positively correlated with the inflammatory responses while being positively or negatively correlated with the immune regulators and immune effectors, respectively. In addition, the same OTUs must display opposite trends in the oHFM group compared with the hHFM group. The summary of the results is displayed in Fig. 8. A total of 33 OTUs (especially, Proteobacteria and Firmicutes OTUs) were identified in the intestinal and respiratory tissues across the three transplantation experiments based on the selection criteria mentioned above. Most of the Proteobacteria OTUs (i.e., Escherichia, *Bilophila*, *Citrobacter*, *Desulfovibrio*, Haemophilus, Proteus, and *Suttrella*) were negatively correlated with the inflammatory responses while being positively correlated with the immune regulators and immune effectors in the hHFM group. On the other hand, most of the Firmicutes OTUs (i.e., *Coprococcus*, *Faecalibacterium*, *Holdemania*, *Paenibacillus*, *Peptococcus*, *Ruminococcus*, and *Selenomonas*) were positively correlated with the inflammatory responses while being negatively correlated with the immune regulators and immune effectors in the hHFM group. *Renibacterium* was also identified several times to be positively correlated with the inflammatory responses while being negatively correlated with the immune regulators and immune effectors in the hHFM group.

## DISCUSSION

Alterations in the host microbiome composition have been associated with various pathological disorders (35). Gut complications and outcome of respiratory infections are linked with abdominal diseases due to strong association between respiratory and intestinal immune axis (36). Influenza virus takes advantage of intestinal dysbiosis by causing robust weakening of beneficial bacterial populations, altering the mucosal immune system, and thus, facilitating secondary bacterial infections in lungs (37). Important differences in gut microbiota composition have been identified between obese and healthy human populations (38). Obese individuals often harbor lower bacterial richness and an imbalanced microbiome, which plays a key role in pathogenesis of obesity (38). Further, obesity may enhance the threat of influenza virus infection, and the duration and transmission of virus shedding (39). Large animal models can be used efficiently to mimic the human respiratory infections like influenza and health conditions such as obesity to better understand the complex interactions between the microbiome, pathogen, host, and immune responses. We previously showed that Gn pig is a suitable animal model conserving the integrity of the HFM transplant thereby allowing a deeper understanding human rotavirus infection and its influence on vaccination (31, 40). In this study, we analyzed the impact of influenza on hHFM and oHFM microbiota in humanized pigs in terms of microbiome composition on the immune system maturation and elucidate the complex interactions between the host immune system-microbiome-influenza. Further, this study provided baseline information about the minimal time required for differential maturation of the immune system during influenza infection using hHFM and oHFM microbiota humanized pig models. Gn pigs not transplanted with any human children microbiota remained free from any microbes in their gut, and when they were infected with influenza suffered from exacerbated disease compared with HFM transplanted experimental animals (data not shown).

In 3-weeks oHFM transplanted piglets reduced challenge virus load in the lungs was observed. In the 5-weeks oHFM transplanted piglets infected with a trend in the reduction of the virus load in the nasal passage (but not in the lungs), was observed which was correlated with an increased pro-inflammatory cytokines IL-12 and TNF-α expression in the TBLN. This result suggests that the longer (5 weeks) the microbes colonize in oHFM humanized pigs, the higher the trigger in the pro-inflammatory mediator secretion to influenza in the respiratory immune system. A study suggested that gut microbiome plays a vital role to response to influenza viral infection (41). Compared with healthy lean mice the obese mice infected with influenza virus exhibited higher lung inflammatory response, lung damage, and viral burden (42). In obese mice, the greater lung damage and delayed expression of the pro-inflammatory cytokines IL-6, TNF-α, and type-I IFNs were induced upon influenza virus infection over the wild-type cohort healthy mice (43). Individuals with obesity have higher circulating lipopolysaccharide (LPS) levels associated with elevated pro-inflammatory cytokines TNF-α and IL-6 expression (44). The types of gut bacteria present in obese people disrupt the gut barrier resulting in LPS entering the systemic circulation from gut and activates production of inflammatory cytokines milieu in the body which, in turn, leads to local inflammation (45). Obese individuals are known to have predominant expression of M1 pro-inflammatory cytokines while the healthy lean populations have activated M2 anti-inflammatory macrophages (46). The obese gut microbes secrete higher LPS which bind to toll-like receptors on immune cells and activate the pro-inflammatory cascades both in the intestines and at distant mucosal sites (47, 48). Likewise, in this Gn piglet study also oHFM transplantation enhanced pro-inflammatory cytokines resulting in inflammatory environment in the body.

The original HFM sample has higher relative abundance of *Prevotella* (10.2%) signature which was absent in the outgrowth samples. A reasonable number of studies demonstrated that increased abundance of *Prevotella* associated with augmented Th17 mediated mucosal inflammation and also responsible for chronic inflammatory disease (49–52). On the other hand, *Faecalibacterium* a healthy human gut microbe (53) present in the original HFM was not identified in the outgrowth sample. More studies are required to further understand these modulations. The oHFM colonized pigs were characterized by lower abundance in Firmicutes, further pathobionts were enriched (Klebsiella and *Akkermansia*) while potential beneficial gut bacteria were reduced (*Bifidobacterium*, *Lactococcus*, *Megasphaera*, *Turicibacter*, and Streptococcus) in the respiratory and intestinal tissues compared with the hHFM pigs. The 2- and 3-weeks oHFM colonized pigs before influenza infection had a significantly higher abundance in Klebsiella compared with hHFM group. These data are consistent with previous studies describing associations between obesity and abundance of Klebsiella (21, 54). Interestingly, the differences observed in the intestinal and respiratory tissues are more pronounced at PCD 7 than at PCD 0 and PCD 2. Similarly, at 3-weeks oHFM colonized animals, *Akkermansia* was in higher abundance in the intestinal and respiratory tissues of oHFM cohorts compared with hHFM cohorts. These differences were also more pronounced over time (at PCD 7 compared with PCD 0 and PCD 2). However, these findings are opposite to previous studies describing a negative association between obesity and Akkermansia muciniphila (55, 56). *A. muciniphila* is a mucin degrading bacterium involved in the gut barrier function homeostasis, antimicrobial peptides production, and possess anti-inflammatory properties (55, 56). On the other hand, in 5-weeks hHFM colonized pigs, the abundance of *Akkermansia* in the respiratory tissues was negatively correlated with inflammatory responses and positively correlated with immune regulators and immune effectors, which supports previous studies promoting the beneficial properties of *Akkermansia* in attenuating viral infection (23, 57–60). By consequence, we hypothesize that the overall microbiome composition may influence the abundance of *Akkermansia* in the oHFM pigs, and thus, its influence on the immune system and influenza viral infection.

The relative abundance of *Bifidobacterium*, *Lactococcus*, *Megasphaera*, *Turicibacter*, and Streptococcus was significantly higher in hHFM pigs compared with the oHFM pigs. Further, the abundance of *Lactococcus* in the intestinal tissues and *Turicibacter* in the intestinal and respiratory tissues was negatively correlated with the immune inflammatory responses, while being positively correlated with immune regulators and immune effectors in hHFM pigs. Similar microbiome profiles were described in influenza-B infected individuals characterized by lower level of *Megasphaera* compared with healthy individuals (61) Oral inoculation of recombinant *Lactococcus* induces mucosal and systemic immunity, and protects mice from influenza virus infection (62). In germ-free mice, oral transplantation of *Bifidobacterium* reduced the severity of influenza virus infection and mortality (10). It was also showed in mice that high-fat diets reduces the abundance of several genera (e.g., *Turicibacter* and *Bifidobacterium*) and positively correlated with the production of butyric acid, a short chain fatty acid (SCFA) with antimicrobial properties (63–65). SCFA are essential component of the gut metabolome contributing to healthier adipocytes, improving energy metabolism, and protection against chronic inflammatory diseases (66–68). A reduced production of SCFA was shown to significantly affect the functions of alveolar macrophages thus increasing lung pathology (37). On the other hand, the role of SCFA during influenza infections is inconsistently reported between studies (37, 67, 69, 70). Other bacteria such as *Lactococcus*, *Megasphaera*, Streptococcus have been showed to produce other SCFA (formate, acetate, and valerate), lactic acids and vitamin B-1 with beneficial impact on the gut homeostasis and host immune system (71–73). Overall, data presented in our study highlights the potential role for SCFA-producing microbiota in modulating obesity and influenza virus infections.

The abundance of *Turicibacter*, Klebsiella, *Akkermansia*, and *Lactococcus* closely correlated between the gut and respiratory tissues, suggesting the gut microbiome composition (rectal swabs, colon and ileum) as well as the colonization duration (2-, 3-, and 5-weeks post-transplantation) have an impact on the microbiome composition of the respiratory (nasal swabs, lung, and BAL fluid) and systemic (MLN) tissues. Microbiota composition modulated by the colonization duration correlated with the immune responses, and the influenza virus infection outcome. In oHFM and hHFM pigs after 5 weeks of transplantation, higher frequencies of T helper/memory cells and cytotoxic T cells with increased correlations between microbiota and immune parameters compared with 2- and 3-weeks post-transplantation animals was observed. In our earlier studies, we revealed that higher frequencies of T helper/memory cells and cytotoxic T cells were associated with the clearance of influenza virus infection in pigs (74–76). The present study highlights the importance of length of colonization of gut microbes in transplanted pigs play a key role in optimal maturation of the microbiome and its interactions with the host immune system. Our finding suggests that a microbial maturation of at least 5 weeks may be necessary for proper maturation of the immune system in Gn pigs to fight against the influenza virus infection.

In conclusion, our study demonstrated that obese HFM transplanted pigs harbored an altered gut microbiome (i.e., Klebsiella and *Akkermansia*) with depleted in SCFA-producing bacteria (i.e., *Bifidobacterium*, *Lactococcus*, *Megasphaera*, *Turicibacter*, and Streptococcus) compared with pigs transplanted with a healthy lean children microbiome. Further, the abundance of these bacteria was also modulated by the colonization duration of the transplant. By consequence, the humanized pig model demonstrated that the length of colonization following microbiome transplantation has an impact on the microbiome maturity, interactions between the host microbiome and its immune system, mucosal immune maturation and the influenza virus infection outcome. We conclude that longer duration of colonization of microbiome (at least 5-weeks post-transplantation) is required in oHFM colonized pigs to reflect the expression of pro-inflammatory cytokines mediated by influenza virus infection like observed typically in obese people. There is a potential of fecal transplants as an avenue to combat obesity.

## MATERIALS AND METHODS

### Collection of human fecal microbiota samples.

The fecal material of obese (*n* = 5) and healthy lean (*n* = 5) children aged 12 to 17 years were collected based on the standard body mass index values. The details of the nature and objective of this study was provided to donor and their parents and obtained a written consent as per the guidelines of The Ohio State University Institutional Review Board. The procedure of sample collection, processing, and storage of aliquots of samples were followed as described previously (31). Briefly, one teaspoon of fresh stool sample was collected in a sterile glass bottle containing 0.1 M potassium phosphate buffer (pH 7.2) and after mixing the fecal materials were stored frozen in 15% glycerol at −80°C.

### Preparation of the inoculum, transplantation of Gn pigs, virus infection, and collection of samples.

In this study, the Gn pigs fecal material outgrowth, prepared by transplanting the Gn pigs with the pooled five obese and five healthy lean children original HFM was used. We used pig fecal material outgrowth in this study because in our initial two studies, most of the original obese children HFM transplanted Gn pigs (approximately 20) became severely sick by day 2 post-transplantation resulting in their early removal from the study. For preparation of the pig outgrowth of human microbiota the entire colon content was collected after 2 weeks of transplantation of two of the survived oHFM transplanted and equal number of hHFM transplanted pigs, pooled separately, suspended in anaerobic medium by vortexing, and stored frozen in 15% glycerol until used in our transplantation experiments describe below.

The care and maintenance of Gn pigs, procedure of fecal outgrowth microbiota transplantation, and swine influenza virus infection was followed as reported earlier (31, 76). Briefly, Cesarean section-derived pigs were maintained in The Ohio State University germ-free facility in sterile bubbles and fed with an infant milk formula. The isolators were sterilized using the SPOR-KLENZ ready to use kit (STERIS, Ohio) before placing newly born piglets. Sterility of pigs was confirmed by culturing rectal swabs on blood agar in both aerobic and anaerobic conditions before HFM colonization as described previously (77, 78). At 2 weeks of age, Gn pigs were administered orally with 40 mL of infant milk formula containing 1 mL of outgrowth healthy lean or obese HFM (hHFM or oHFM), respectively. Transplanted pigs were infected intranasally with 6 × 10^6^ TCID_50_ of a virulent swine influenza virus H1N1 (A/Swine/OH/24366/2007) (31, 75) 2-, 3-, or 5-weeks post-transplantation. Each experiment consisted of two groups (hHFM and oHFM; 4 to 6 pigs per group). Pigs were monitored twice daily for clinical flu signs (fever, lethargy, anorexia, and labored breathing). Nasal and fecal swab samples from pigs were collected at 0-, 2-, 4-, and 7-PCD for microbiome analysis and to determine the viral load. On the day of necropsy (PCD 7), BAL fluid, blood for isolating peripheral blood mononuclear cells (PBMCs), and tracheobronchial lymph nodes (TBLN), and mesenteric lymph nodes (MLN) tissues for isolating mononuclear cells (MNCs) were collected as described earlier (76). TBLN and MLN samples were also collected in *RNAlater* for gene expression analysis and BAL, lung, ileum, colon, and MLN samples were collected for microbiome analysis (Fig. 1).

### Titration of influenza virus, flow cytometry analysis and quantitative reverse transcription-PCR (qRT-PCR) analysis.

All these analyses were performed by following the procedures described previously by us (74–76). Briefly, nasal and BAL fluid samples were subjected to virus titration using Madin-Darby Canine Kidney epithelial (MDCK) cells. Samples treated plates were incubated for 36 h at 37°C in 5% CO_2_ incubator, immunostained with influenza virus nucleoprotein specific primary antibody (CalBioreagents, CA) followed by Alexa Fluor 488 conjugated goat anti-mouse IgG (H+L) secondary antibody (Life Technologies, CA). The signals were read under a fluorescence microscope and virus titers were calculated as described (76).

The isolated PBMCs and MNCs of TBLN and MLN were immunostained for T-helper/memory cells (CD3^+^CD4^+^CD8α^+^β-), cytotoxic T lymphocytes (CTLs) (CD3+CD4-CD8α^+^β^+^), and myeloid cells (CD172^+^) using specific immune markers as described previously (31, 76, 79). The immunostaining procedure and gating pattern are described in our earlier studies (31, 76, 79) and the same was followed for this study. The immunostained cells were fixed, and 100,000 events were acquired by using the BD Aria II flow cytometer (BD Biosciences, CA) and the data were analyzed using FlowJo software (Tree Star, Palo Alto, CA). The specific anti-pig and respective isotype control monoclonal antibodies used were fluorochrome labeled such as CD3 (PerCP), CD4α (APC/Cy7), CD8α (FITC), monocyte/granulocyte CD172 (PE) (Southernbiotech, AL); CD8ß (PE-Cy7) (BD Biosciences, CA).

TBLN and MLN tissues were processed using *TRIzol^R^* reagent (Invitrogen, Carlsbad, CA) and 2 μg of extracted total RNA was used to synthesize cDNA (80). The internal control β-actin and target genes IFNγ, TNF-α, IL-6, and IL-12 (Table S1) mRNA expression were performed using the Green Supermix kit (Bio-Rad Laboratories, CA) by qRT-PCR (Applied Biosystems, CA) and the fold changes in target gene expression were calculated as described previously (81, 82).

### Genomic DNA extraction, sequencing, and bioinformatics analyses.

Genomic DNA from the rectal and nasal swabs, and BAL fluid was extracted using the *PureLink Microbiome DNA* purification kit (Invitrogen, Carlsbad, CA), and DNeasy blood and tissue kit (Qiagen, Hilden, Germany) was used to extract the DNA from the pig’s respiratory (lung), systemic (MLN), and intestinal (colon and ileum) tissues (78). The quality of the extracted DNA was assessed using nanodrop.

The 16S rRNA (V4-V5 region) was amplified using Phusion High-Fidelity PCR Kit (New England Biolabs Inc, Ipswich, MA), PCR products were cleaned using AMPure XP PCR kit (Beckman Coulter Inc, Beverly MA), and sequenced using Illumina MiSeq 300-base paired-end kit, as described previously (78). Similar extraction procedures using sterile water was used as control for 16S RNA sequencing to account for environmental contamination that might occur during the sample processing steps. Quality control and processing of the raw reads was performed using FastQC (83), Trimmomatic (84). Trimmed reads were analyzed in Quantitative Insights into Microbial Ecology (QIIME) software version 2 (85) using SILVA 16S reference data set (138.1 release; arb-silva.de). Ninety six percent of the sequences (*n* = 369/383) met the desired quality (min of 100 counts for nasal and BAL samples and 1,000 counts for intestinal and fecal samples) and were considered for further analysis of the microbiota (alpha and beta diversity analysis).

### Statistical analyses.

The use of 4 to 6 pigs per group per time point allowed sufficient statistical power (power of 0.857 with CI of 95%) to detect significant differences between the groups. The statistical comparisons were made between oHFM and hHFM groups for a designated time point, samples type and type of experiment throughout the study unless specified otherwise by using JMP Pro 15 (SAS; Cary, NC), GraphPad Prism 5 (San Diego, CA), and Rstudio (Boston, CA). The virus titer, mRNA expression, and flow cytometry data were analyzed by using *unpaired t test*. The data were presented as the mean of 4 to 6 pigs ± SEM. Asterisks denote significant difference (*, *P* < 0.05; **, *P* < 0.01; ***, *P* < 0.001).

For microbiota analyses, a Kruskal Wallis combined with a Dunn’s *post hoc* test was performed to identify significant differences in Shannon index. Principal coordinate analysis (PCoA) combined with a permutational multivariate analysis of variance (PERMANOVA) and the measurement of the entropy R^2^ was used to study the spatial clusterization of the beta diversity data (unweighted and weighted uniFrac values). An analysis of composition of microbes (ANCOM) combined with a Bootstrap Forest model were used to identify differences in relative abundance between the phylum and species level. A *P* value ≤0.01 and LogWorth[FDR] >2 were used to determine the statistical significance of the microbiota data. The similarities in microbiota composition at the genus level was used to estimate the “stability” of microbiota between the original HFM, the pig outgrowth HFM, and the tissues collected at PCD 0 and PCD 7 for the designated pig group. OTUs were considered as “inconsistent” if they were detected in less than 50% of the samples of a designated group.

A multivariate statistical analysis (wide range Pearson correlation coefficient) was performed to identify significant correlations (*P* > 0.01 and r^2^ lower than −0.25 or higher than 0.25) at PCD 7 between the microbiota data (at the genus/species level) and the immune response data associated with inflammatory responses (TNF-α, IL-6, and IFNγ), immune regulators (IL-12 and CD3-CD172^+^), and immune effectors (CD3^+^CD4^+^CD8α^+^β- and CD3+CD4-CD8α^+^β+T cells). The microbiota data showing significant correlations with the immune parameters were selected to build a scoring system allowing to select only the microbiota/immune combinations showing opposite trends between the hHFM and the oHFM groups. Only the microbiota/immune combinations showing opposite trends between HFM groups were selected for further analysis. A two-way clustering and multiple correspondence analyses were used to separate the microbiota based on their correlations with the immune parameters. In addition, the following criteria were used to identify the OTUs likely to be associated with immune maturation, implicated with influenza infection or with obesity induced inflammation in our HFM pig model, because more differences were seen at the OTU level than at the phylum or genus level. In hHFM group, the selected OTUs were negatively correlated with the inflammatory responses while being positively correlated with the immune regulators and immune effectors, and the opposite trend was observed in oHFM group.
