# Supplementary material for: Gut Microbiota of Obese Children Influences Inflammatory Mucosal Immune Pathways in the Respiratory Tract to Influenza Virus Infection: Optimization of an Ideal Duration of Microbial Colonization in a Gnotobiotic Pig Model
Source: Microbiol Spectr. 2022 May 17;10(3):e02674-21. doi: 10.1128/spectrum.02674-21 (PMC9241774; doi:10.1128/spectrum.02674-21)
Supplement: SUPPLEMENTAL FILE 1 — Supplemental material. Download spectrum.02674-21-s0001.pdf, PDF file, 1.1 MB [file spectrum.02674-21-s0001.pdf]

SUPPLEMENTAL MATERIAL

Supplemental FIG 1 Microbiota similarity at the genus level between the original human fecal microbiota (HFM) and the pig outgrowth HFM collected. Only the OTUs with an average relative abundance higher than 0.1% are displayed in this table. N= 1 fecal sample per group. Mean RA: average relative abundance across the four HFM groups for the designated OTU. Red cells: OTUs detected in the designated sample. Blue cells: OTUs not detected in the designated sample.

Supplemental FIG 2 Microbiome correlations between sample types for a designated length of transplantation and transplant type. **A)** Beta diversity differences between sample types across all time points and length of transplantation. Intestinal: rectal swab, colon and ileum samples; Systemic: MLN; Respiratory: nasal swab, lung, and BAL. **B)** Microbiome composition similarity profile between 2-, 3-, and 5-weeks post-transplantation at the genus/species levels. Numbers in the circles indicate the number of OTUs correlated across different tissue types (n=61 OTUs total). **C)** OTUs correlated between the sample types. Res, Int and Sys: respiratory, intestinal, and systemic tissues; RS & NS: rectal and nasal swabs. O & H: Obese and healthy human fecal microbiome transplants.

Supplemental Figure 1

OTUs	Mean RA	Healthy HFM		Obese HFM	
		Original	Outgrowth	Original	Outgrowth
Bacteroides	12.1%				
Prevotella	10.2%				
Akkermansia	9.4%				
Verrucomicrobia	9.4%				
Bifidobacterium	4.8%				
Streptococcus	2.4%				
Enterococcus	2.3%				
[*Ruminococcus*]	2.0%				
Klebsiella	1.6%				
Faecalibacterium	1.4%				
Blautia	1.0%				
Methanobrevibacter	0.9%				
Methanobacteria	0.9%				
Corynebacterium	0.8%				
[*Eubacterium*]	0.7%				
Ruminococcus	0.6%				
Dorea	0.6%				
Collinsella	0.6%				
Parabacteroides	0.5%				
Lactococcus	0.4%				
Phascolarctobacterium	0.4%				
Lactobacillus	0.4%				
Coprococcus	0.3%				
Clostridiaceae	0.3%				
Clostridium	0.3%				
Oscillospira	0.3%				
Staphylococcus	0.3%				
Megamonas	0.2%				
Pyramidobacter	0.2%				
Dialister	0.1%				
Anaerotruncus	0.1%				
Turicibacter	0.1%				
**% similarity with original hHFM**		**100.0%**	**99.9%**	99.9%	99.5%
**% similarity with outgrowth hHFM**		**95.4%**	**100.0%**	85.0%	89.7%
**% similarity with original oHFM**		98.8%	89.6%	**100.0%**	**99.0%**
**% similarity with outgrowth oHFM**		93.7%	97.4%	**95.0%**	**100.0%**

Supplemental Figure 2

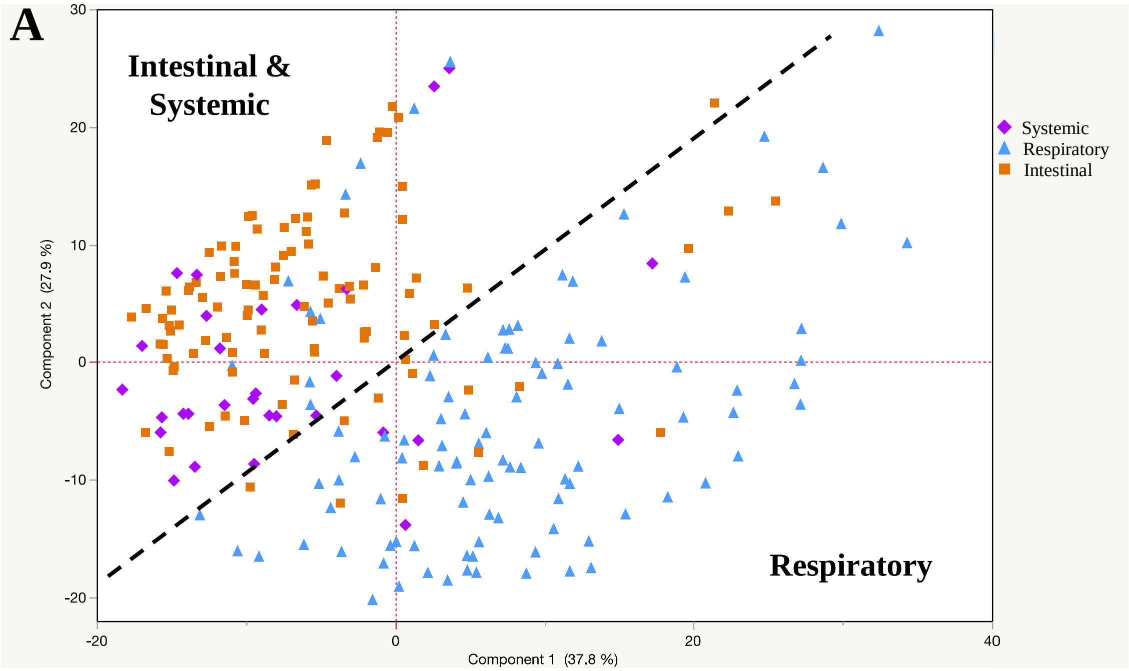

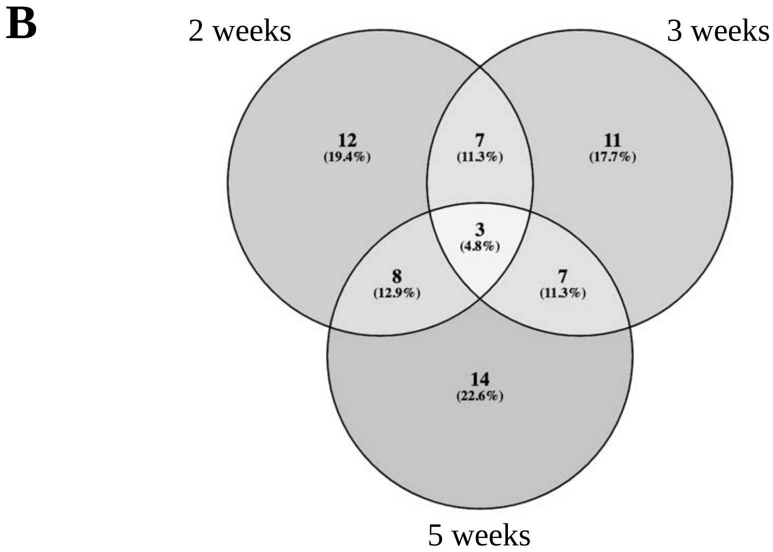

Supplemental Figure 2

C

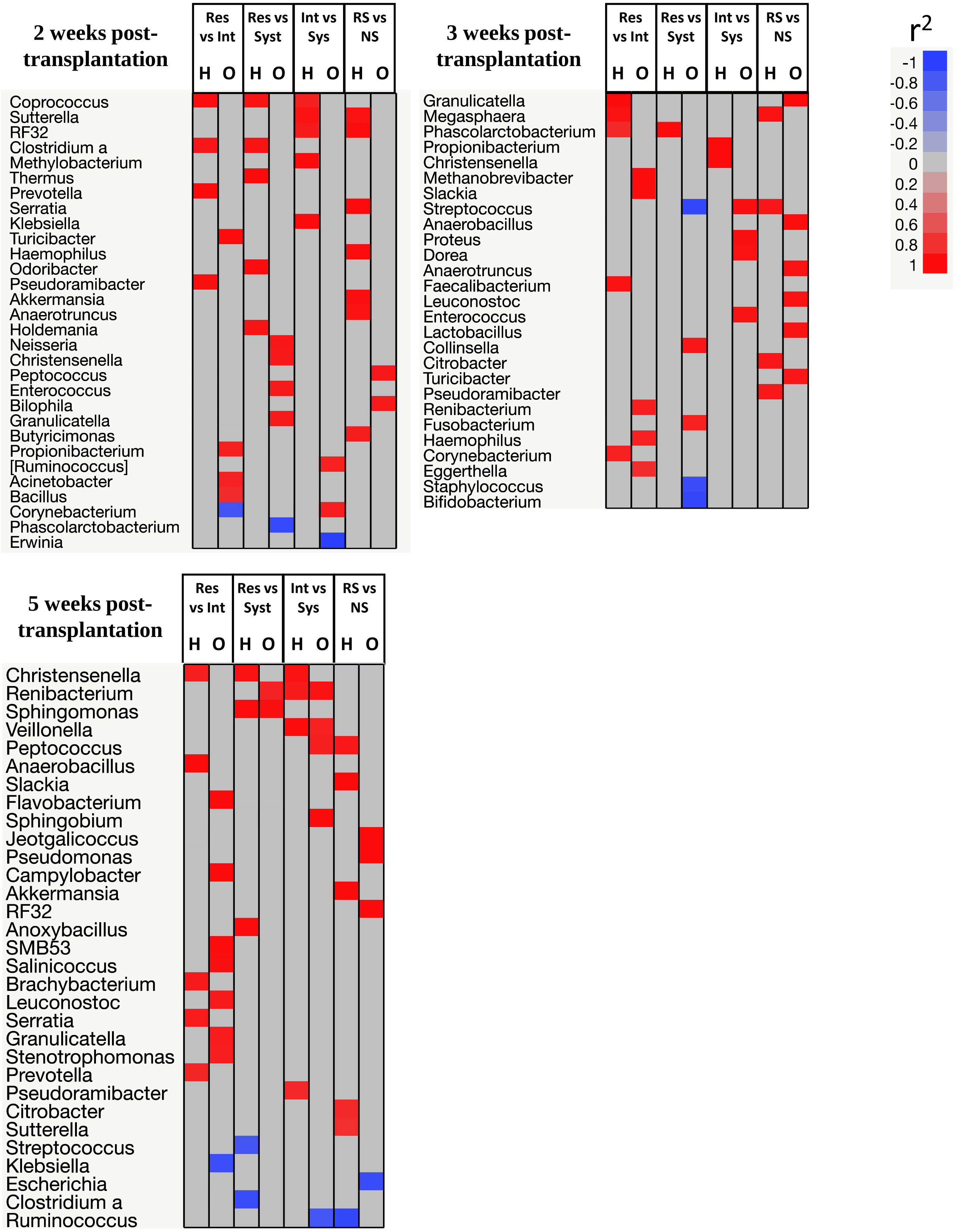

Supplemental Table 1 Sequences of the primers used in qRT-PCR analyses.

Supplemental Table 2 Differences in alpha and beta diversity between healthy HFM and obese HFM pig samples. Values indicate the means and standard deviations in parenthesis. Cells highlighted in gray and bold indicates significant differences ($P<0.01$). NS: nasal swab; RS: rectal swab; BAL: Bronchoalveolar lavage fluid; MLN: mesenteric lymph node. Nasal and rectal swabs were collected at post-challenge day 0 (A0), 2 (A2), and 7 (A7). Intestinal (ileum and colon), respiratory (lung and BAL) and systemic tissues (MLN) were collected at post-challenge day 7 (PCD7).

Supplementary Table 3 Relative abundance of OUT between hHFM and oHFM transplanted pig samples. Significant differences ($P<0.01$) in relative abundance detected between hHFM and oHFM for a designated time post transplantation, time point post influenza challenge and sample type. O & H: Obese and healthy human fecal microbiome transplants.

Supplemental Table 1

S.No	Oligo name	Sequence (5’→3’)
1	*β-actin*	CAGCCTCCTGAAACTGGAATAT (F)<br>TCAGCAACAAGGTCTACAATCC (R)
2	*TNF-α*	CGTTGTAGCCAATGTCAAAGCC (F)<br>TGCCCAGATTCAGCAAAGTCCA (R)
3	*IL-12*	GATGCTGGCCAGTACACC (F)<br>TCCAGCACGACCTCAATG (R)
4	*IFN-γ*	CAGCTTTGCGTGACTTTGTG (F)<br>GATGAGTTCACTGATGGCTTT (R)
5	*IL-6*	CCAGGAACCCAGCTATGAAC (F)<br>CTGCACAGCCTCGACATT (R)

Supplemental Table 2

Experiment	Time point	Tissue	Alpha diversity (Shannon index)			Beta diversity (weighted uniFrac)	
			Healthy	Obese	P value	r^2 entropy	P value
HFM pigs challenged at 2 weeks post-challenge	A0	NS	**5.36 (0.16)**	4.73 (0.52)	<0.0001	0.5	<0.0001
		RS	**4.84 (0.32)**	4.54 (0.5)	0.0003	0.8	<0.0001
	A2	NS	3.18 (1.14)	**3.92 (0.43)**	<0.0001	1	<0.0001
		RS	**5.01 (0.29)**	4.35 (0.68)	<0.0001	1	<0.0001
	A7	NS	2.99 (0.56)	**4.28 (0.63)**	<0.0001	1	<0.0001
		BAL	4.65 (0.38)	4.68 (0.32)	0.65	0.92	<0.0001
		Lung	4.09 (0.67)	**4.91 (0.26)**	<0.0001	0.66	<0.0001
		RS	4.14 (0.54)	**4.74 (0.39)**	<0.0001	0.86	<0.0001
		Colon	3.63 (0.28)	**4.68 (0.3)**	<0.0001	0.57	<0.0001
		Ileum	3.4 (1.27)	3.41 (0.92)	0.94	0.81	<0.0001
		MLN	4.67 (0.32)	4.76 (0.29)	0.13	1	<0.0001
HFM pigs challenged at 3 weeks post-challenge	A0	NS	3.3 (0.85)	**3.81 (0.34)**	<0.0001	1	<0.0001
		RS	**4.28 (0.74)**	3.87 (0.46)	0.0004	1	<0.0001
	A2	NS	4.33 (0.5)	**4.52 (0.21)**	0.007	1	<0.0001
		RS	**5.04 (0.25)**	4.37 (0.46)	<0.0001	1	<0.0001
	A7	NS	3.94 (0.32)	**4.63 (0.4)**	<0.0001	1	<0.0001
		BAL	4.88 (0.2)	**5.16 (0.32)**	<0.0001	0.68	<0.0001
		Lung	**4.86 (0.42)**	3.89 (0.72)	<0.0001	0.77	<0.0001
		RS	**4.49 (0.49)**	3.69 (0.33)	<0.0001	1	<0.0001
		Colon	3.85 (0.73)	4.0 (0.52)	0.17	1	<0.0001
		Ileum	3.94 (0.7)	**4.7 (0.27)**	<0.0001	1	<0.0001
		MLN	4.73 (0.15)	4.82 (0.35)	0.18	0.54	<0.0001
HFM pigs challenged at 5 weeks post-challenge	A0	NS	3.53 (0.59)	3.81 (0.61)	0.014	1	<0.0001
		RS	5.17 (0.37)	**5.36 (0.21)**	0.0011	1	<0.0001
	A2	NS	3.35 (0.48)	**3.89 (0.74)**	0.0094	0.07	
		RS	4.83 (0.49)	4.99 (0.41)	0.061	0.92	<0.0001
	A7	NS	4.31 (0.34)	4.08 (0.8)	0.0421	0.68	<0.0001
		BAL	3.31 (0.97)	**4.99 (0.41)**	<0.0001	1	<0.0001
		Lung	3.9 (1.16)	3.9 (0.72)	0.97	0.98	<0.0001
		RS	**4.42 (0.32)**	3.59 (0.5)	<0.0001	1	<0.0001
		Colon	4.22 (0.3)	4.29 (0.35)	0.25	0.98	<0.0001
		Ileum	3.75 (0.54)	**4.3 (0.55)**	<0.0001	0.9	<0.0001
		MLN	3.6 (1.39)	**4.54 (0.31)**	<0.0001	0.8	<0.0001

Table 3

Incubation time between human fecal microbiome transplantation and influenza challenge (weeks post-transplantation)	Time point (day post-influenza challenge)	Tissue type	Operational taxonomic unit (from phyla to species levels)	p-Value	hHFM mean	hHFM standard deviation	oHFM mean	oHFM standard deviation
3	2	Rectal swab	[Barnesiellaceae]	0.0051	0.00324429	0.00279389	0.00034375	0.00021158
2	7	MLN	[Mogibacteriaceae]	0.0097	7.2116E-05	0.00016126	0.01047605	0.01900387
3	7	Rectal swab	[Mogibacteriaceae]	0.0051	0.00030951	0.00017098	5.0717E-05	2.5028E-05
2	2	Nasal swab	[Odoribacteraceae]	0.0067	0.00000334	7.4685E-06	0.00088785	0.00132589
3	7	Colon	4C0d-2	0.0096	0.00033473	0.00053899	0	0
5	2	Rectal swab	4C0d-2	0.0062	6.1833E-06	1.5146E-05	0.00020984	0.00019167
3	0	Rectal swab	Actinobacteria	0.0051	0.06058177	0.06373382	0.00056801	0.00046216
3	2	Rectal swab	Actinobacteria	0.0051	0.0626754	0.046781	0.00134379	0.00111792
3	0	Rectal swab	Aeromonadaceae	0.0028	0	0	0.00075427	0.00041489
3	2	Rectal swab	Aeromonadaceae	0.0028	0	0	0.00046317	0.00024774
3	7	Colon	Aeromonadaceae	0.0048	0.00000865	1.1289E-05	0.00020724	0.0001357
3	7	Rectal swab	Aeromonadaceae	0.0043	0.00000935	1.4721E-05	0.00043912	0.00010483
3	0	Rectal swab	Aeromonadales	0.0028	0	0	0.00075427	0.00041489
3	2	Rectal swab	Aeromonadales	0.0028	0	0	0.00046317	0.00024774
3	7	Colon	Aeromonadales	0.0048	0.00000865	1.1289E-05	0.00020724	0.0001357
3	7	Rectal swab	Aeromonadales	0.0043	0.00000935	1.4721E-05	0.00043912	0.00010483
3	0	Nasal swab	Akkermansia	0.0051	0.0005045	0.00047121	0.06643207	0.08773369
3	0	Rectal swab	Akkermansia	0.005	0.00045306	0.00060376	0.44106827	0.16765562
3	2	Nasal swab	Akkermansia	0.0051	0.00040088	0.00040509	0.08999828	0.0502755
3	2	Rectal swab	Akkermansia	0.0051	0.00059801	0.00060325	0.34306428	0.10107744
3	7	Colon	Akkermansia	0.0051	0.00123183	0.00224897	0.37470405	0.14651604
3	7	Ileum	Akkermansia	0.0051	0.00692983	0.01503791	0.09661962	0.02981109
3	7	Nasal swab	Akkermansia	0.0081	0.00082716	0.00066552	0.08605315	0.03512945
3	7	Rectal swab	Akkermansia	0.0051	0.00022955	0.00034635	0.47680078	0.11858566
3	0	Rectal swab	Alcaligenaceae	0.0028	0.00236992	0.00273668	0	0
3	2	Nasal swab	Alcaligenaceae	0.0096	0.00041166	0.00032137	0	0
3	2	Rectal swab	Alcaligenaceae	0.0028	0.00437081	0.00615812	0	0
3	7	Colon	Alcaligenaceae	0.0037	0.00324214	0.00320787	0.00000245	6.0012E-06
3	7	Ileum	Alcaligenaceae	0.0028	0.00362943	0.0077101	0	0
3	7	Rectal swab	Alcaligenaceae	0.0028	0.00221325	0.00351803	0	0
3	7	Colon	Alphaproteobacteria	0.0028	0.00016953	0.00017604	0	0
3	7	Rectal swab	Alphaproteobacteria	0.0028	0.00011494	8.6346E-05	0	0
2	7	Nasal swab	Bacillales	0.0081	0.59802397	0.1276531	0.18661993	0.06736629
2	7	Nasal swab	Bacilli	0.0081	0.779025	0.10178727	0.30458922	0.13672629
3	0	Rectal swab	Bacilli	0.0051	0.0873187	0.12529997	0.0041331	0.0023965
3	2	Rectal swab	Bacilli	0.0051	0.09472182	0.06744344	0.00587676	0.00209399
3	7	Ileum	Bacilli	0.0051	0.3533066	0.21906408	0.111941	0.04543133
3	7	Rectal swab	Bacilli	0.0082	0.07066964	0.06917422	0.01330533	0.00824781
2	7	Nasal swab	Bacillus	0.008	0.00026147	0.00011214	0.00004205	3.2935E-05
3	2	Rectal swab	Bacteroidaceae	0.0082	0.17179743	0.11384417	0.03477983	0.02298768
3	7	Ileum	Bacteroidaceae	0.0082	0.00948835	0.01738109	0.09161746	0.04661626
3	2	Rectal swab	Bacteroidales	0.0082	0.29134622	0.1003966	0.08335358	0.04372637
3	2	Rectal swab	Bacteroides	0.0082	0.17179743	0.11384417	0.03477983	0.02298768
3	7	Ileum	Bacteroides	0.0082	0.00948835	0.01738109	0.09161746	0.04661626
3	2	Rectal swab	Bacteroidetes	0.0082	0.29134622	0.1003966	0.08335358	0.04372637
3	2	Rectal swab	Bacteroidia	0.0082	0.29134622	0.1003966	0.08335358	0.04372637
3	0	Rectal swab	Betaproteobacteria	0.0051	0.00252077	0.00269851	0.00011349	5.1201E-05
3	2	Nasal swab	Betaproteobacteria	0.005	0.0005987	0.00024096	0.00010927	0.00012082
3	2	Rectal swab	Betaproteobacteria	0.0051	0.00508503	0.00606947	7.2468E-05	4.8229E-05
3	7	Colon	Betaproteobacteria	0.0082	0.00352276	0.00350674	0.0000366	2.6216E-05
3	7	Rectal swab	Betaproteobacteria	0.0051	0.00269824	0.00358687	0.00011614	6.3499E-05
3	0	Nasal swab	Bifidobacteriaceae	0.0051	0.01999388	0.00997961	0.00438772	0.00150019
3	0	Rectal swab	Bifidobacteriaceae	0.0051	0.06046455	0.06371865	0.00043369	0.00045879
3	2	Rectal swab	Bifidobacteriaceae	0.0051	0.06217253	0.04634009	0.0010885	0.00120388
3	0	Nasal swab	Bifidobacteriales	0.0051	0.01999388	0.00997961	0.00438772	0.00150019
3	0	Rectal swab	Bifidobacteriales	0.0051	0.06046455	0.06371865	0.00043369	0.00045879
3	2	Rectal swab	Bifidobacteriales	0.0051	0.06217253	0.04634009	0.0010885	0.00120388
3	0	Nasal swab	Bifidobacterium	0.0051	0.01999388	0.00997961	0.00438772	0.00150019
3	0	Rectal swab	Bifidobacterium	0.0051	0.06046455	0.06371865	0.00043369	0.00045879
3	2	Rectal swab	Bifidobacterium	0.0051	0.06217253	0.04634009	0.0010885	0.00120388
5	7	Lung	Bilophila	0.0096	0	0	0.00013936	0.00013166
5	7	MLN	Bilophila	0.0072	1.4033E-05	2.2048E-05	0.00035753	0.00039754
3	7	Nasal swab	Blautia	0.0081	0.00077787	0.00038524	0.00516888	0.00155187
5	0	Rectal swab	Blautia	0.0081	0.01255538	0.02305846	0.00052949	0.000669
3	0	Rectal swab	Burkholderiales	0.0051	0.00252077	0.00269851	0.00011349	5.1201E-05

3	2	Nasal swab	Burkholderiales	0.0081	0.00053601	0.00026692	0.00010129	0.00011967
3	2	Rectal swab	Burkholderiales	0.0051	0.00508503	0.00606947	7.2468E-05	4.8229E-05
3	7	Colon	Burkholderiales	0.0051	0.00352077	0.003503	0.00003255	2.3269E-05
3	7	Rectal swab	Burkholderiales	0.0051	0.00269824	0.00358687	0.00011614	6.3499E-05
3	2	Nasal swab	Campylobacter	0.0081	0.00052426	0.00063846	5.0086E-05	4.8381E-05
3	2	Nasal swab	Campylobacteraceae	0.0081	0.00052426	0.00063846	5.0086E-05	4.8381E-05
3	2	Nasal swab	Campylobacterales	0.0081	0.00052426	0.00063846	5.0086E-05	4.8381E-05
5	0	Nasal swab	Carnobacteriaceae	0.008	6.8351E-05	5.9315E-05	0.00001026	9.4816E-06
5	7	Lung	Carnobacteriaceae	0.0096	0	0	0.00011526	0.00019181
3	2	Rectal swab	Christensenella	0.0051	0.0021154	0.00219838	0.00019429	0.00013429
3	2	Rectal swab	Christensenellaceae	0.0051	0.00224035	0.00216372	0.00023965	0.00016472
3	0	Nasal swab	Citrobacter	0.005	3.2533E-05	2.8623E-05	0.00316872	0.00129702
3	0	Rectal swab	Citrobacter	0.0028	0	0	0.00110419	0.0007145
3	2	Rectal swab	Citrobacter	0.0043	2.0583E-05	3.5635E-05	0.00122917	0.00045591
3	7	Colon	Citrobacter	0.0051	5.8555E-05	7.312E-05	0.00096753	0.00066418
3	7	Ileum	Citrobacter	0.0037	4.1667E-06	1.0206E-05	0.00023776	8.7207E-05
3	7	Rectal swab	Citrobacter	0.0043	2.9644E-05	6.4643E-05	0.00066082	0.00022144
3	0	Rectal swab	Clostridiaceae	0.0051	0.00947222	0.00305484	0.0011476	0.00110441
3	2	Rectal swab	Clostridiaceae	0.0051	0.01803121	0.01196224	0.00170377	0.00146716
3	0	Rectal swab	Clostridium	0.0051	0.00722661	0.00209252	0.00083229	0.00091248
3	2	Rectal swab	Clostridium	0.0082	0.01343452	0.00961278	0.00130434	0.00127314
3	7	Rectal swab	Clostridium	0.0082	0.00195053	0.00169218	0.00016041	0.00015407
3	2	Rectal swab	Collinsella	0.0051	0.00343023	0.00179431	0.00019654	0.00020591
3	7	Rectal swab	Coprococcus	0.0082	0.00053486	0.00036379	6.6674E-05	7.0757E-05
2	2	Rectal swab	Coriobacteriaceae	0.0097	0.00067072	0.00130295	0.00000586	1.3103E-05
3	2	Rectal swab	Coriobacteriaceae	0.0051	0.00360695	0.00173112	0.00021673	0.00022484
2	2	Rectal swab	Coriobacteriales	0.0097	0.00067072	0.00130295	0.00000586	1.3103E-05
3	2	Rectal swab	Coriobacteriales	0.0051	0.00360695	0.00173112	0.00021673	0.00022484
2	2	Rectal swab	Coriobacteriia	0.0097	0.00067072	0.00130295	0.00000586	1.3103E-05
3	2	Rectal swab	Coriobacteriia	0.0051	0.00360695	0.00173112	0.00021673	0.00022484
3	0	Rectal swab	Deltaproteobacteria	0.0082	0.00772009	0.00446453	0.00212333	0.00135689
3	2	Nasal swab	Desulfovibrio	0.0072	0.00388722	0.0042111	0.0000094	1.6112E-05
3	2	Rectal swab	Desulfovibrio	0.0043	0.00492693	0.00157812	1.6317E-05	2.5346E-05
3	7	Colon	Desulfovibrio	0.0082	0.00687028	0.00640012	0.000024	1.6615E-05
3	7	Ileum	Desulfovibrio	0.0048	0.00051493	0.00070968	0.00001625	1.9864E-05
3	7	Rectal swab	Desulfovibrio	0.0043	0.00688651	0.00615915	0.0000079	1.2271E-05
3	2	Nasal swab	Dorea	0.005	0.00125399	0.00084362	7.7402E-05	9.6976E-05
3	7	Rectal swab	Dorea	0.0051	0.00780798	0.00651579	0.00012199	0.00017369
2	7	Rectal swab	Eggerthella	0.0067	0.00052071	0.00056994	0.0000053	8.4131E-06
3	2	Nasal swab	Eggerthella	0.0051	0.00058559	0.00024524	0.00010209	0.00010738
3	2	Rectal swab	Eggerthella	0.0096	0.00017671	0.00031106	0	0
3	7	Nasal swab	Eggerthella	0.0081	0.00175197	0.00096769	0.00033085	0.00037974
3	0	Rectal swab	Enterococcaceae	0.0051	0.00261836	0.00144714	0.0004325	0.00025813
3	2	Rectal swab	Enterococcaceae	0.0082	0.00616393	0.00326764	0.00059437	0.00049265
3	0	Rectal swab	Enterococcus	0.0051	0.0021433	0.00125658	0.00035698	0.00022142
3	2	Rectal swab	Enterococcus	0.0082	0.00516605	0.00307468	0.0004943	0.00041003
3	2	Nasal swab	Epsilonproteobacteria	0.0081	0.00052426	0.00063846	5.0086E-05	4.8381E-05
3	0	Nasal swab	Erwinia	0.0043	0.00000655	1.0565E-05	0.00013568	8.3835E-05
3	0	Rectal swab	Erwinia	0.0043	3.3463E-05	5.757E-05	0.00119929	0.0007189
3	2	Rectal swab	Erwinia	0.005	0.00003295	2.7869E-05	0.00106235	0.00074801
3	7	Colon	Erwinia	0.0051	0.00003225	2.2544E-05	0.00045846	0.00014877
3	7	Rectal swab	Erwinia	0.005	6.0891E-05	7.047E-05	0.00081509	0.00026801
5	7	Lung	Erwinia	0.0096	0	0	8.5707E-05	0.00014715
3	2	Nasal swab	Escherichia	0.0051	0.00140832	0.00042845	0.00029892	0.00016946
3	2	Rectal swab	Eubacteriaceae	0.0081	0.00030773	0.00034398	5.0777E-05	5.1783E-05
5	7	Lung	Eubacteriaceae	0.0096	0	0	0.00178553	0.00212325
5	2	Rectal swab	Euryarchaeota	0.0096	0.003466	0.00347339	0	0
2	7	Nasal swab	Firmicutes	0.0081	0.80632454	0.08828479	0.37634597	0.16788225
3	7	Ileum	Firmicutes	0.0051	0.74132093	0.18288701	0.28760108	0.08283994
3	7	Rectal swab	Firmicutes	0.0051	0.24735335	0.12036784	0.05543628	0.03088007
5	7	Lung	Granulicatella	0.0096	0	0	0.00010528	0.00019391
5	0	Rectal swab	Holdemania	0.0048	0.00027686	0.00023462	1.0203E-05	1.3945E-05
2	2	Nasal swab	Klebsiella	0.0081	0.00351452	0.0069551	0.10040632	0.1194458
2	7	Nasal swab	Klebsiella	0.0081	0.00810442	0.00968557	0.11006179	0.11333551
3	0	Nasal swab	Klebsiella	0.0051	0.00022697	0.00019134	0.14276306	0.07912362
3	0	Rectal swab	Klebsiella	0.005	6.1723E-05	5.0713E-05	0.12024103	0.08587382
3	2	Nasal swab	Klebsiella	0.0051	0.00087968	0.00130134	0.12613955	0.05389586
3	2	Rectal swab	Klebsiella	0.005	5.675E-05	6.2667E-05	0.1189844	0.06399903
3	7	Colon	Klebsiella	0.0051	0.00045341	0.00047141	0.10026263	0.05689266
3	7	Ileum	Klebsiella	0.0051	0.00204839	0.0045744	0.13388641	0.0609416
3	7	Nasal swab	Klebsiella	0.0081	0.00205309	0.00114867	0.0842068	0.04738297
3	7	Rectal swab	Klebsiella	0.0051	0.00017305	0.00020798	0.11435351	0.06793483
2	2	Nasal swab	Lachnospiraceae	0.0081	0.00236523	0.00196199	0.02232798	0.01159099

3	7	Nasal swab	Lactobacillaceae	0.0081	0.00989852	0.00373583	0.02166638	0.00711943
3	0	Rectal swab	Lactobacillales	0.0082	0.07832957	0.12339577	0.00328716	0.00204052
3	2	Rectal swab	Lactobacillales	0.0051	0.07769875	0.06458075	0.00485096	0.00193082
3	7	Nasal swab	Lactobacillus	0.0081	0.00989852	0.00373583	0.02166638	0.00711943
2	2	Rectal swab	Lactococcus	0.0097	0.01723674	0.01633612	0.00000326	7.2896E-06
2	7	BAL	Lactococcus	0.0097	0.01811956	0.0298693	0.00019889	0.00048717
2	7	Ileum	Lactococcus	0.0067	0.00437749	0.00468123	9.9333E-06	1.5661E-05
2	7	MLN	Lactococcus	0.0075	0.00671091	0.0060974	0	0
2	7	Nasal swab	Lactococcus	0.0081	0.09832954	0.02836197	0.00086582	0.00066537
2	7	Rectal swab	Lactococcus	0.0081	0.01019744	0.00573752	5.0224E-05	6.7404E-05
3	0	Nasal swab	Lactococcus	0.0048	0.01951547	0.015072	0.0000252	3.0233E-05
3	0	Rectal swab	Lactococcus	0.0028	0.00408309	0.00658011	0	0
3	2	Nasal swab	Lactococcus	0.0048	0.0305157	0.031488	4.8467E-05	9.5493E-05
3	2	Rectal swab	Lactococcus	0.0037	0.00160281	0.00179152	9.6833E-06	2.3719E-05
3	7	Colon	Lactococcus	0.005	0.0037091	0.00345467	2.2927E-05	2.4471E-05
3	7	Nasal swab	Lactococcus	0.0081	0.02481673	0.00177834	0.00020338	0.00022477
3	7	Rectal swab	Lactococcus	0.005	0.00329298	0.0028445	0.00002235	2.4333E-05
2	7	Nasal swab	Leuconostocaceae	0.0055	0.0039468	0.00146379	8.0833E-06	1.98E-05
3	0	Nasal swab	Leuconostocaceae	0.005	0.02666041	0.019035	9.3513E-05	0.00013212
3	2	Nasal swab	Leuconostocaceae	0.005	0.01196286	0.00768295	0.00019618	0.00021886
3	2	Rectal swab	Leuconostocaceae	0.0096	8.1634E-05	6.5048E-05	0	0
3	7	Nasal swab	Leuconostocaceae	0.0081	0.00445563	0.00161842	0.00018168	0.00031502
2	2	Rectal swab	Megasphaera	0.0075	0.01769388	0.0233084	0	0
2	7	Rectal swab	Megasphaera	0.0055	0.01075852	0.01529379	3.0667E-06	7.5118E-06
3	0	Nasal swab	Megasphaera	0.0028	0.00028927	0.00049298	0	0
3	2	Rectal swab	Megasphaera	0.0096	0.00133082	0.00175526	0	0
5	2	Rectal swab	Methanobacteria	0.0096	0.003466	0.00347339	0	0
5	2	Rectal swab	Methanobacteriaceae	0.0096	0.003466	0.00347339	0	0
5	2	Rectal swab	Methanobacteriales	0.0096	0.003466	0.00347339	0	0
5	2	Rectal swab	Methanobrevibacter	0.0096	0.003466	0.00347339	0	0
2	2	Nasal swab	Odoribacter	0.0067	0.00000334	7.4685E-06	0.00028544	0.00040649
3	2	Rectal swab	Odoribacter	0.0081	0.00226328	0.00398578	6.3782E-05	9.5437E-05
2	2	Nasal swab	Oscillospira	0.008	5.3772E-05	6.8188E-05	0.00200078	0.00145986
3	0	Nasal swab	Pasteurellaceae	0.0062	0.00001245	3.0496E-05	0.00352135	0.00459493
3	7	Ileum	Pasteurellaceae	0.0096	0	0	0.00352406	0.00530982
3	0	Nasal swab	Pasteurellales	0.0062	0.00001245	3.0496E-05	0.00352135	0.00459493
3	7	Ileum	Pasteurellales	0.0096	0	0	0.00352406	0.00530982
5	7	Lung	Peptococcaceae	0.0096	0	0	0.00138272	0.0024018
5	7	Lung	Peptococcus	0.0096	0	0	0.00138272	0.0024018
3	0	Nasal swab	Peptostreptococcaceae	0.005	0.00063336	0.00035815	3.6611E-05	3.9566E-05
3	0	Rectal swab	Peptostreptococcaceae	0.0028	0.00045948	0.00056575	0	0
3	2	Nasal swab	Peptostreptococcaceae	0.0043	0.00340772	0.00145665	0.0000108	2.0748E-05
3	2	Rectal swab	Peptostreptococcaceae	0.0028	0.0009795	0.0007224	0	0
3	7	Colon	Peptostreptococcaceae	0.005	0.00028315	0.00023381	1.3517E-05	1.182E-05
3	7	Ileum	Peptostreptococcaceae	0.0051	0.04910578	0.04076949	0.00089273	0.00080476
3	7	Nasal swab	Peptostreptococcaceae	0.0075	0.00324701	0.00161157	9.8884E-05	0.00014342
3	7	Rectal swab	Peptostreptococcaceae	0.0043	0.00039534	0.00022378	0.00002915	4.5819E-05
2	0	Rectal swab	Phascolarctobacterium	0.0093	0.00577012	0.00660463	0.00019207	0.00050817
3	2	Nasal swab	Phascolarctobacterium	0.0096	0.00132792	0.00160478	0	0
3	7	Ileum	Phascolarctobacterium	0.0096	0.00047434	0.0010529	0	0
5	7	MLN	Phyllobacteriaceae	0.0096	0.00224656	0.00171269	0	0
2	7	Nasal swab	Planococcaceae	0.0081	0.00139645	0.00041972	0.00051913	0.00016519
2	7	Nasal swab	Proteobacteria	0.0081	0.11527354	0.07133634	0.43883535	0.25248327
3	2	Rectal swab	Pseudoramibacter	0.0081	0.00030773	0.00034398	5.0777E-05	5.1783E-05
5	7	Lung	Pseudoramibacter	0.0096	0.00000514	1.1493E-05	0	0
3	2	Nasal swab	RF32	0.0096	5.574E-05	5.3636E-05	0	0
3	7	Colon	RF32	0.0096	0.00016456	0.00018095	0	0
3	7	Ileum	RF32	0.0096	0.00107738	0.00213139	0	0
3	7	Rectal swab	RF32	0.0028	0.00010927	7.579E-05	0	0
5	7	Ileum	Rhizobiaceae	0.0096	0	0	0.00201618	0.00232555
2	2	Nasal swab	Ruminococcaceae	0.0081	0.00112401	0.00174447	0.00775698	0.00226345
5	0	Rectal swab	Ruminococcaceae	0.0051	0.02264665	0.01436014	0.00449798	0.00417763
5	0	Nasal swab	Ruminococcus	0.0081	0.00048004	0.0003126	0.00220549	0.00119902
5	7	Colon	Ruminococcus	0.0096	0.00443513	0.00586171	0	0
3	0	Nasal swab	Slackia	0.0096	0	0	0.000114	0.00010447
3	2	Nasal swab	Slackia	0.0096	0	0	0.00015866	0.00012148
3	2	Rectal swab	SMB53	0.0028	8.9077E-05	6.9649E-05	0	0
3	7	Ileum	SMB53	0.0096	7.9633E-05	4.493E-05	0	0
2	7	Nasal swab	Staphylococcaceae	0.0081	0.58894089	0.12686104	0.18377879	0.06775844
2	7	Nasal swab	Staphylococcus	0.0081	0.58883546	0.12681194	0.18374797	0.06774337
2	7	Nasal swab	Streptococcaceae	0.0081	0.15012341	0.02470549	0.04828394	0.03402166
3	0	Nasal swab	Streptococcaceae	0.0082	0.05543715	0.01712487	0.01975219	0.00812362
3	0	Rectal swab	Streptococcaceae	0.0082	0.06922725	0.11247352	0.00250115	0.00162037

3	2	Nasal swab	Streptococcaceae	0.0051	0.14117844	0.04762141	0.05527084	0.01567714
3	2	Rectal swab	Streptococcaceae	0.0051	0.0645173	0.05986551	0.00309249	0.00134113
3	7	Ileum	Streptococcaceae	0.0051	0.12842103	0.04510881	0.02679388	0.00652975
3	7	Lung	Streptococcaceae	0.0081	0.12588923	0.07269488	0.02634101	0.02182142
3	7	Nasal swab	Streptococcaceae	0.0081	0.143395	0.06005188	0.04030941	0.00528788
3	7	Rectal swab	Streptococcaceae	0.0051	0.06411819	0.06989517	0.00617463	0.00436546
3	2	Rectal swab	Streptococcus	0.0051	0.06291449	0.06099093	0.0030828	0.00135547
3	7	Ileum	Streptococcus	0.0051	0.12531216	0.04195387	0.02634643	0.00680356
3	7	Lung	Streptococcus	0.0081	0.12553	0.07243565	0.02634101	0.02182142
3	7	Nasal swab	Streptococcus	0.0081	0.11857827	0.06120164	0.04010335	0.00529521
3	7	Rectal swab	Streptococcus	0.0051	0.06082521	0.07068762	0.00615228	0.00435203
3	0	Rectal swab	Sutterella	0.0028	0.00236992	0.00273668	0	0
3	2	Nasal swab	Sutterella	0.0096	0.00041166	0.00032137	0	0
3	2	Rectal swab	Sutterella	0.0028	0.00437081	0.00615812	0	0
3	7	Colon	Sutterella	0.0037	0.00324214	0.00320787	0.00000245	6.0012E-06
3	7	Ileum	Sutterella	0.0028	0.00362943	0.0077101	0	0
3	7	Rectal swab	Sutterella	0.0028	0.00221325	0.00351803	0	0
3	7	Rectal swab	Trabulsiella	0.0096	0	0	0.0000257	1.5076E-05
3	0	Rectal swab	Turicibacter	0.0051	0.00808041	0.00314732	0.00021724	0.00020121
3	2	Nasal swab	Turicibacter	0.0082	0.01887604	0.01143314	0.00185663	0.00156147
3	2	Rectal swab	Turicibacter	0.0051	0.01522489	0.00742987	0.00038249	0.00056573
3	7	Rectal swab	Turicibacter	0.0051	0.00070628	0.00042488	0.00011013	0.00010975
3	0	Rectal swab	Turicibacteraceae	0.0051	0.00808041	0.00314732	0.00021724	0.00020121
3	2	Nasal swab	Turicibacteraceae	0.0082	0.01887604	0.01143314	0.00185663	0.00156147
3	2	Rectal swab	Turicibacteraceae	0.0051	0.01522489	0.00742987	0.00038249	0.00056573
3	7	Rectal swab	Turicibacteraceae	0.0051	0.00070628	0.00042488	0.00011013	0.00010975
3	0	Rectal swab	Turicibacterales	0.0051	0.00808041	0.00314732	0.00021724	0.00020121
3	2	Nasal swab	Turicibacterales	0.0082	0.01887604	0.01143314	0.00185663	0.00156147
3	2	Rectal swab	Turicibacterales	0.0051	0.01522489	0.00742987	0.00038249	0.00056573
3	7	Rectal swab	Turicibacterales	0.0051	0.00070628	0.00042488	0.00011013	0.00010975
3	2	Nasal swab	Veillonella	0.0078	3.0005E-05	4.7774E-05	0.00786589	0.01828268
3	7	BAL	Veillonella	0.0067	9.8083E-05	0.00019197	0.00208798	0.00084992
3	0	Nasal swab	Verrucomicrobia	0.0051	0.0005045	0.00047121	0.06643207	0.08773369
3	0	Rectal swab	Verrucomicrobia	0.005	0.00045306	0.00060376	0.44106827	0.16765562
3	2	Nasal swab	Verrucomicrobia	0.0051	0.00040088	0.00040509	0.08999828	0.0502755
3	2	Rectal swab	Verrucomicrobia	0.0051	0.00059801	0.00060325	0.34306428	0.10107744
3	7	Colon	Verrucomicrobia	0.0051	0.00123183	0.00224897	0.37470405	0.14651604
3	7	Ileum	Verrucomicrobia	0.0051	0.00693438	0.01503611	0.0969187	0.02945493
3	7	Nasal swab	Verrucomicrobia	0.0081	0.00082716	0.00066552	0.08605315	0.03512945
3	7	Rectal swab	Verrucomicrobia	0.0051	0.00022955	0.00034635	0.47680078	0.11858566
3	0	Nasal swab	Verrucomicrobiaceae	0.0051	0.0005045	0.00047121	0.06643207	0.08773369
3	0	Rectal swab	Verrucomicrobiaceae	0.005	0.00045306	0.00060376	0.44106827	0.16765562
3	2	Nasal swab	Verrucomicrobiaceae	0.0051	0.00040088	0.00040509	0.08999828	0.0502755
3	2	Rectal swab	Verrucomicrobiaceae	0.0051	0.00059801	0.00060325	0.34306428	0.10107744
3	7	Colon	Verrucomicrobiaceae	0.0051	0.00123183	0.00224897	0.37470405	0.14651604
3	7	Ileum	Verrucomicrobiaceae	0.0051	0.00693438	0.01503611	0.0969187	0.02945493
3	7	Nasal swab	Verrucomicrobiaceae	0.0081	0.00082716	0.00066552	0.08605315	0.03512945
3	7	Rectal swab	Verrucomicrobiaceae	0.0051	0.00022955	0.00034635	0.47680078	0.11858566
3	0	Nasal swab	Verrucomicrobiae	0.0051	0.0005045	0.00047121	0.06643207	0.08773369
3	0	Rectal swab	Verrucomicrobiae	0.005	0.00045306	0.00060376	0.44106827	0.16765562
3	2	Nasal swab	Verrucomicrobiae	0.0051	0.00040088	0.00040509	0.08999828	0.0502755
3	2	Rectal swab	Verrucomicrobiae	0.0051	0.00059801	0.00060325	0.34306428	0.10107744
3	7	Colon	Verrucomicrobiae	0.0051	0.00123183	0.00224897	0.37470405	0.14651604
3	7	Ileum	Verrucomicrobiae	0.0051	0.00693438	0.01503611	0.0969187	0.02945493
3	7	Nasal swab	Verrucomicrobiae	0.0081	0.00082716	0.00066552	0.08605315	0.03512945
3	7	Rectal swab	Verrucomicrobiae	0.0051	0.00022955	0.00034635	0.47680078	0.11858566
3	0	Nasal swab	Verrucomicrobiales	0.0051	0.0005045	0.00047121	0.06643207	0.08773369
3	0	Rectal swab	Verrucomicrobiales	0.005	0.00045306	0.00060376	0.44106827	0.16765562
3	2	Nasal swab	Verrucomicrobiales	0.0051	0.00040088	0.00040509	0.08999828	0.0502755
3	2	Rectal swab	Verrucomicrobiales	0.0051	0.00059801	0.00060325	0.34306428	0.10107744
3	7	Colon	Verrucomicrobiales	0.0051	0.00123183	0.00224897	0.37470405	0.14651604
3	7	Ileum	Verrucomicrobiales	0.0051	0.00693438	0.01503611	0.0969187	0.02945493
3	7	Nasal swab	Verrucomicrobiales	0.0081	0.00082716	0.00066552	0.08605315	0.03512945
3	7	Rectal swab	Verrucomicrobiales	0.0051	0.00022955	0.00034635	0.47680078	0.11858566
3	0	Nasal swab	Weissella	0.0028	0.00054532	0.00032414	0	0
3	2	Nasal swab	Weissella	0.0028	0.00021477	0.00013447	0	0
3	7	Colon	YS2	0.0096	0.00033473	0.00053899	0	0
5	2	Rectal swab	YS2	0.0062	6.1833E-06	1.5146E-05	0.00020984	0.00019167
